# A decision support model for efficient garment reprocessing for a sustainable circular business model

**DOI:** 10.1007/s00170-025-17164-5

**Published:** 2025-12-25

**Authors:** Hariketan Patel, Gokula Vasantha, Jonathan Corney, John Quigley, Hanane El-Raoui, Rachel Sales, Michael Cusack, Anthony Burns, Andrew Rough

**Affiliations:** 1 Edinburgh Napier University, Edinburgh, EH10 5DT UK; 2 University of Edinburgh, Edinburgh, EH8 9YL UK; 3 University of Strathclyde, Glasgow, G1 1XQ UK; 4 ACS Clothing LTD, Motherwell, ML1 4GP UK

**Keywords:** Open jackson queueing network, Decomposition, Tradeoff curves, Rental-based circular garment business model, Sustainability

## Abstract

The rapidly growing fashion business has intensified environmental, and sustainability concerns due to its focus on high-volume production at low costs. The rental-based business models in the circular economy, by promoting an alternative approach to garment ownership, aim to mitigate environmental impact, enhance businesses’ competitiveness, and provide user value to customers. The rapid reprocessing of garment rentals offered to customers boosts corporate earnings and mitigates environmental issues. However, considering the complexities of garment reprocessing and fluctuations in rental demand, the objective of this study is to use an open Jackson queueing network with a decomposition approach to analyse the effects of external arrival rates and worker’s processing rates on lead processing time and throughput for different garment types. The tradeoff curves provide a sensitivity analysis by varying the external arrival rate and the worker’s processing rate to examine their impact on lead time and throughput of garment types. The results based on the workcell utilization demonstrate the tradeoff in throughput of various garment types by varying process parameters in the garments reprocessing. The results indicate that deviations in process parameters beyond certain upper and lower thresholds result in reduced throughput and increased lead time for garment types. The rearrangement of workers in the reprocessing workcell network showed a favourable improvement in the throughput of garment types. The findings significantly benefit the garment sector by offering a methodical approach for efficient processing, consequently improving overall efficiency and sustainability, and promoting circular economy efforts.

## Introduction

The textile industry has experienced rapid growth due to global outsourcing and supply chain innovations, and contributes significantly to the global economy with around $1.3 trillion and over 300 million employees along the value chain [1]. The decline in price rates and rapid delivery of garments to the customer, the total number of garments purchased per person in the EU has risen by 45% in the last decade, and over 100 million new garments have been produced globally, and this number keeps increasing with continuous customer demand [2]. The cultivation of raw materials like cotton requires a large volume of water and pesticides. The fabric production consumes large amounts of energy, water, chemicals, and resources to convert raw materials into fibres and fabrics by spinning, followed by weaving and dyeing [3]. The global textile sector generates more than 1 billion tons of CO_2_ equivalent, exceeding the overall emissions from international aviation and maritime transport [4]. The inexpensive garments are often discarded after a few uses, leading to substantial consumption of virgin resources and detrimental environmental impacts, including greenhouse gas emissions, excessive water consumption, deforestation, hazardous chemical usage, soil degradation, and landfilling [5]. A throwaway culture and excessive purchasing have led to the early dumping of textile waste and garments, culminating in more than 14 million tons of waste being deposited in landfills. The reduction in both the frequency and amount of garment consumption, and the transition from purchasing tangible garments to intangible services, can mitigate negative impacts and achieve sustainability. The life span and sustainable use of clothing are critical to reducing waste and greenhouse gas emissions [6]. Consequently, sustainability in the clothing industry has become a significant focus for researchers.

The modern sustainable economy aims to minimize value depletion and degradation while enhancing resource effectiveness and value growth. To facilitate a comprehensive revolution in the clothing sector, creative thinking, advanced technology, novel materials, and novel business strategies are essential. The novel business structures with diverse value-generating modalities are essential for effective execution [7, 8]. The linear business model of the garment industry has four distinct stages: (1) Harvest natural resources, (2) Take (transport of raw material), (3) Make (Garment manufacturing), and (4) Dispose (dumping waste garments in landfills) [9]. The linear garment industry exploits several natural resources to produce low-cost, low-quality garments. Consumers utilize these clothes for a short time before dumping them. The increased production of clothing leads to less usage, creating a significant waste issue and posing challenges for society and green sustainability [10]. The circular (sustainable) garment business model concept focuses on minimizing waste and enhancing the utilization of resources by reusing the garments before dumping. The circular model will yield innovative, sustainable business models capable of decoupling environmental damage from economic growth. The extent to which industry activities involving waste and consumption, facilitated by circular business models, may minimize the environmental impact of the linear model has been the primary focus of research on the circular economy in the fashion industry [11]. The different circular garment business models may include:

1. Extended product lifecycle: Addressing marketing issues and introducing measures such as repairing, transforming, swapping, or reselling on second-hand markets can extend the useful life of goods in their initial state and purposeful use, hence reducing waste [12].
2. Circular resources: adopting advanced garment processing and utilization by substituting virgin materials with recyclable and reusable materials, hence eliminating linear resources [12].
3. Resource reuse: Apply a novel recycling method to convert the original waste product into a new product. For this, take back facilities are provided in the business model [13].
4. Product-as-a-service: Replace conventional ownership-based business structure with a pay-to-use or rental contract, allowing business firms to preserve ownership. The rental business model enables customers to retain garments for a limited time. The subscription model allows customers to acquire a variety of clothing based on a monthly or annual subscription [14].

The circular business model encounters several problems, including financial, technological, organizational, institutional, market, and value chain obstacles. Financial challenges may adversely affect a business’s viability, stability, or profitability. Economic challenges, especially for SMEs, restrict the implementation of circular business models. Initial investment costs, including research & development, materials, and logistics, may escalate swiftly [15]. Technological challenges include a lack of investment in technology, poor labour skills, and weak management [16]. Organizational challenges include uncertainty, dependence on linear approaches, discrepancies between the circular-based business model and the initial timeline, and possible expertise limits [17]. Conventional fashion enterprises have difficulties in implementing sustainable business practices owing to insufficient customer interest and awareness [18]. Implementing circular processes in the fashion business is difficult owing to variables such as insufficient client awareness, loyalty, supply chain complications, and inadequate network support [19].

In various surveys for opinions and behaviours about clothing rentals, specifically focusing on the various types of garments, people prefer to rent rather than buy. The results reveal that 33% of participants rent party gowns, 2% lease pre-wedding dresses, and 59% get wedding clothing via rental. 79.6% of respondents express satisfaction with rental clothing services, indicating that these services fulfil their needs for short-term garments. Individuals choose to rent clothes for several reasons, including minimizing environmental impact, utilizing premium garments, saving financial resources, reducing wardrobe space, and presenting an elegant look for any event. 72% of respondents said that trendy clothes, designer brand items, affordable rates, and event-appropriate clothing are the primary motivators for renting garments. 66% of individuals rarely rent an outfit, 32% do so rarely, and 2% do so two to three times a year. Customers must consider factors like insurance, flexible rental duration, dry cleaning, cleanliness, ease of pickup and return, and competitive pricing and fees when choosing clothing rental services. Among those using clothes rental services, 64.6% deemed all these characteristics significant [20].

The business model for clothing rental services comprises three steps. First, retrieve the rented clothes from the client. Second, garment reprocessing includes washing, repairs, ironing, and repacking. Third, deliver the reprocessed garments to the consumer upon demand. Many challenges exist in garment reprocessing to achieve cleanliness, comply with governmental regulations, and treat garments according to type, colour, material, and size. Each item is precisely inspected throughout processing to eliminate any stains, followed by appropriate washing and ironing. Each garment demanded different treatment according to the quality of the garment, as returned by the customer. The demand for garments may vary throughout the year due to seasonal changes, weddings, and holidays. To maximize profit, it is essential for the garment reprocessing industry to properly process garments and prepare them for customer rental. Therefore, it is necessary to analyse the impact of various scenarios in the garment processing business to enhance reprocessing and meet customer demands. This will enhance the profit in the garment rental business model.

### Challenges and problems

The demand for different garment types (Jacket, Trousers, Waistcoat, Kilt, Dresses) fluctuates throughout the year due to several factors, including bank holidays, wedding seasons, weekend events, Christmas festivities, and seasonal demand shifts. All these effects change the garment rental demand and the return garments from the customers, resulting in fluctuations in the external and internal arrival rates of the processing network. Customer demand for various garment types fluctuates owing to these variables, which consequently affect the prioritization of garment processes, hence impacting transit times between the workcells and increasing the complexity of the process network. The addition of new employees with a lower processing rate or the removal of proficient workers from the workcell might impact the overall worker’s processing rate (items/hour). Efficient training of the new employee may improve the worker’s processing rate. The cumulative effect of these variable factors affects the total lead processing time and throughput of each garment type. Consequently, a process manager must use a proficient technological approach to analyse and evaluate the complexities of the processing domain to make decisions that meet the customer’s rental needs.

This article addresses the impact of several operational variables, such as external arrival rate, worker’s processing rate, and the number of workers at each workcell, on total lead time (hours) and throughput for each garment type. We use open Jackson queuing network theory to evaluate the overall lead time and throughput for each garment type by analysing the tradeoff curves via variations in the external arrival rate and the workers’ processing rate, which influence the lead time and throughput of each garment type.

## Literature survey

The problem of interest concerns the study of network queues. The focus is on the flow of items being processed in a factory, where multiple types of items arrive to be cleaned. These items move through the factory, being serviced at various stations, each designed for a specific function. The route taken through the factory depends on the type of item being processed. The aim is to develop modelling approaches to explain the duration of time that items spend in the system, to identify opportunities for improving productivity.

Modelling approaches can range from deterministic methods, such as System Dynamics (SD), to stochastic methods, depending on the extent to which variation in performance is critical for evaluating the system. A popular class of stochastic models for network queues is based on Markovian assumptions [21]. These approaches rely on specific assumptions about item arrival rates and processing times at each station, allowing for the derivation of closed-form mathematical expressions for system performance based on its structural characteristics. However, the Markovian assumptions are not always appropriate [22], hence, they may not fully reflect garment processing routines. In such cases, more general modelling frameworks have been developed to relax these assumptions. These more flexible approaches typically require computer-based simulation to estimate performance measures, with Discrete Event Simulation (DES) being a common choice. Discrete event simulation is a robust method to represent most manufacturing simulations, integrating complex decision rules and stochastic tasks [23]. The computational cost, set-up time, and multiple simulation runs are the major limitations to predicting accurate performance metrics, while an open Jackson queueing network model offers a strategy for critical performance measures, including queue length, resource utilization, and waiting time.

Petri nets in a production facility provide a graphical model of workflow in a complex network. Petri nets are excellent in assessing fundamental characteristics such as connectivity, dynamism, and work in progress [24]. Petri nets need to be transformed into formal frameworks to perform quantitative analysis, which restricts their analytical capabilities for performance measurement compared to an open Jackson queueing network. Machine Learning models are effective for pattern recognition and predictive analytics in manufacturing setups but lack interpretability and causal reasoning for process planning [25]. It required a big structured dataset for efficient predictions. Open Jackson queueing network Models are favored for their analytical simplicity, while Closed Queuing Networks are more suitable for limited task systems but elevate computing complexity. G/G/1 Models provide accurate depictions of variability but do not possess closed-form solutions. Simulation-based methodologies need substantial computing resources and effort. Petri Nets facilitate the modeling of simultaneous processes; however, they are limited to qualitative analysis. System Dynamics Models capture long-term activity but lack precision [26].

Open Queueing frameworks have several applications [27]. Owoloko et al. aim to reduce patient waiting times at the Hospital Center by implementing the open Jackson queuing network and implementing additional staff [28]. Kleinrock’s [29] study on open Jackson queueing network, Wein’s [30] and Shantanukumar and Xu [31] performed approximation analyses on the open Jackson queueing network for generalized networks for simultaneous capacity and demand allocations. Djatmiko and Cahya [32] implemented an open Jackson queueing network in the analysis of the vehicle queue at Suroboyo Carniva. Ratnasari et al. [33] applied Open Jackson queue network in a textile packaging company to reduce packaging time. Bronshtein and Gertsbakh [34] investigated a Jackson-type open exponential queuing network with finite buffers, assuming Poisson flow. They conducted an analytical investigation utilizing nonlinear equations, demonstrating that the findings are consistent with simulations for high connectedness topologies. Open Jackson queueing network offers an analytical framework for evaluating performance metrics in a complex system. Its modularity aligns with complex networks, making it suitable for analysing waiting time, bottlenecks, and support capacity planning.

Queue theory is a network system concept that explains the dynamics of individuals entering, waiting, and departing a network. The performance matrix in queueing networks includes queue length, waiting time, processing time, server utilization, output, and lead processing time [23]. Queueing network models include Feedforward Queueing Network, Gordon-Newell Queueing Network, BCMP (Baskett, Chandy, Muntz, and Palacios) Queueing Network, MVA-MIX (Mean Value Analysis for Mixed networks), FES (Flow Equivalent Server), Jackson Queueing Network, SCAT (Self-Correcting Approximation Technique), BJB (Balanced Job Bounds), closing methods, ABA (Asymptotic Bound Analysis), SUM (Summation method), BOTT (BOTTAPROX method), etc. Queue networks are categorized into three types: open, closed, and mixed queue networks. Examples of open networks include BCMP, ABA, Jackson queue network, and BJB. The mixed network comprises MVA-MIX and closure techniques. FES, BOTT, and ABA are examples of closed network models. The few examples of application of the network model are to serve any service provider (hospital, pharmacy), supply chain management, and manufacturing units [23, 24].

The Jackson queueing network is a system in which individuals (products) may transition between workstations before exiting the network. The system exhibits a Poisson distribution at arrival and an exponential service time. The product transfer has the probability of moving to the subsequent node after being serviced at the preceding node in the network [35, 36] The service rate is characterized as a critical variable in a single-server queue network (M/M/1 or M/G//1). Conversely, the service rate is expressed with the number of servers at the node defined in the multi serving queue network (M/M/m or M/G/m) [37]. Shi [38] enhanced the queue network analysis approach by using a linear approximation for generalized networks. This method employs a heuristic strategy to convert non-linear network balancing equations into linear systems, considering each node as a GI/G/m/k system. Burten et al. [39] used Jackson queue network to evaluate the worker and capacity needed for the manufacturing network. Alexander et al. [40] shown the advantages of using queuing models as a first stages to provide critical data to estimate lead time, work in progress and resource utilization. Table 1 highlights the studies on queueing network models across various industrial sectors.

**Table 1:** Review on queueing network models in different manufacturing areas

References	Service model	Scope of study
Chen and Zhou [41]	M/M/1	Calculate WIP with layout changes
Tu and Chang [42]	M/M/1	Determine the bottleneck and queue time
Zhu and Wu [43]	M/M/m	Determine the optimal design considering vehicle blocking
Nazzal [44],	M/G/m	Estimate throughput capacity
Govind et al. [45].	M/M/m	Calculate WIP and queue delays
Zhou et al. [46].	M/M/1	Calculate WIP considering priority rules
Xu et al. [47].	M/M/m	Determine system design and resource allocation
Chen et al. [48].	M/M/1	Estimate performance measurements
Liao et al. [49].	M/M/m	Identify performance measurements considering uncertainty
Mohammadi et al. [50].	M/M/1	Estimate product cycle time
Zhang et al. [51].	M/M/m	Estimate performance measurements

The manufacturing process network performance model should account for the uncertainty of job demand and waiting queue due to resource unavailability. The theory of queuing networks is widely used for approximation, performance evaluation, and system modelling [52]. Decomposition methods are widely used approximation techniques for open queueing network analysis, which simplify network scale by analysing each sub-network independently [53]. The decomposition method anticipates that distinct queueing networks are statistically independent GI/G/1 queues and that the method for input for each GI/G/1 queue can be represented by a renewed process [54]. The queue estimates derived from decomposition approaches are approximate because they assume the independence of queueing systems, neglecting the substantial influence of arrival stream correlations on performance metrics [55]. Jain and Grassmann [56] applied these criteria to an open queue network, in which the renewal process is approximated by an incoming stream of a subsequent queue. Fu and Kaku [57] developed an open network M/M/c/1 utilizing constrained assumptions, including exponential distribution and infinite queues for product-form predictions. Senthlkumar [58] investigate the consequences of single unidirectional flow operations on the performance of approximations across varying waiting intensities. Benjaafar [59] adopted an open network for transporters and processing facilities. To determine the system work in progress, he implemented the decomposition approach of the Queueing Network Analysis, assuming infinite queues, generalized service time distributions, and idle travel times. Sagron et al. [60] propose a multi-class network to reduce tandem queue downtime, hence reducing bottlenecks at subsequent network nodes and reducing departure uncertainty.

To maintain competitiveness, companies must prioritize sectors for the allocation of time and resources. However, to accommodate budgetary constraints, businesses must make trade-offs. These trade-offs may include opting between uniformity and specialization of products, regulating budget and quality, or deciding between internal production and outsourcing. Companies must evaluate several elements to attain their objectives, and the extent of these trade-offs may vary [61]. Boyer and Lewis [62] show the significance of trade-offs in demonstrating that production capacity can integrate the competitive objectives of expenses, quality, adaptability, and delivery. Nand et al. [61] highlights the growing environmental consciousness and the need for sustainable practices, making it difficult for manufacturing companies to balance their operations and outputs. Bitran [63] explains tradeoff analysis, how the work in progress, in relation to lead time, describes the effect on capacity utilization. A critical issue in managing projects is the time-cost tradeoff problem, which aims to balance the expenses, lead time, and production volume. It is categorized into discrete and continuous models. The discrete model considers many performance modes with distinct expenses and time, whereas the continuous model establishes a consistent integration of time and cost [64].

The literature shows effective implementation of the open Jackson queueing network in minimizing queue network waiting time in manufacturing processes. However, these outcomes cannot be directly extended to the garment reprocessing industry because service capabilities are chosen from a finite set in connection with the fluctuating garment rental demand. Garment reprocessing involves the transfer of garments between the workcells based on consumer quality standards. It entails distinct treatments for each garment, complicating the processing network. Processing managers must make decisions based on variations in input and worker’s processing rates to ready clothing for customer rental. This work, filling the existing literature gap, presents the application of open Jackson queueing network to evaluate the tradeoff in the garment reprocessing by varying the effect of external arrival rate and worker’s processing rate to analyse any garment reprocessing network.

## Objectives

In line with the literature survey and the challenges defined in the previous section, the objective of the work is to explore the open Jackson queueing network model to generate trade-off curves and its use to study the sensitivity analysis of various input parameters and their impact on the overall lead time and throughput of each garment type. To evaluate performance measures, we use the decomposition of the open Jackson queueing network into an independent node model. To evaluate the utilization of each workcell, throughput, and lead time for each garment type during a single day, with all workers working an 8-hour shift. We focus on the tradeoff between (i) variations in arrival rate to the lead time (hours), and throughput (items per hour) for each garment type. (ii) Variations in worker processing speed to lead time (hours) and throughput (items per hour) for each garment type. Finally, keep the initial input data of processing rate and arrival rate the same and rearrange workers based on work cell utilization to see how this arrangement affects the overall lead time and throughput of the current network output.

Consequently, the key contributions of the present work may be summarized as:

- Featuring a scenario-based adaptive open Jackson queueing network model in the garment reprocessing network to evaluate lead time and throughput of different garment types, varying parameters resulting from fluctuating garment rental demand.
- Evaluating the effects of the external arrival rate, worker’s processing rate in relation to the extent of the workcell utilization imposed on the garment reprocessing lead time and throughput.
- Tradeoff analysis of lead time and throughput of different garment types reprocessing, by varying external arrival rate and worker’s processing rate.

The rest of the paper is organized as follows: In Sect. 4, we provide a description of garment reprocessing data. In Sect. 5, we provide an open Jackson queueing network mathematical model and decomposition algorithm. Sect. 6 contains results and discussion. Sect. 7 discusses the arrangement of workers based on the utilization of the work cell. In Sect. 8, we conclude the research and outline potential pathways of further investigation.

## Garment reprocessing data description

A one-year garment reprocessing data is provided by one of the UK’s most reputed garment reprocessing company. They record all processing and movement data of different garment types using barcode attached to each garment. The following information is captured for every scan: barcode, worker ID, workcell, garment type, date time scan-In, date time-Out, colour of garment, size, quality, and time spent in seconds for processing.

**Fig. 1:**
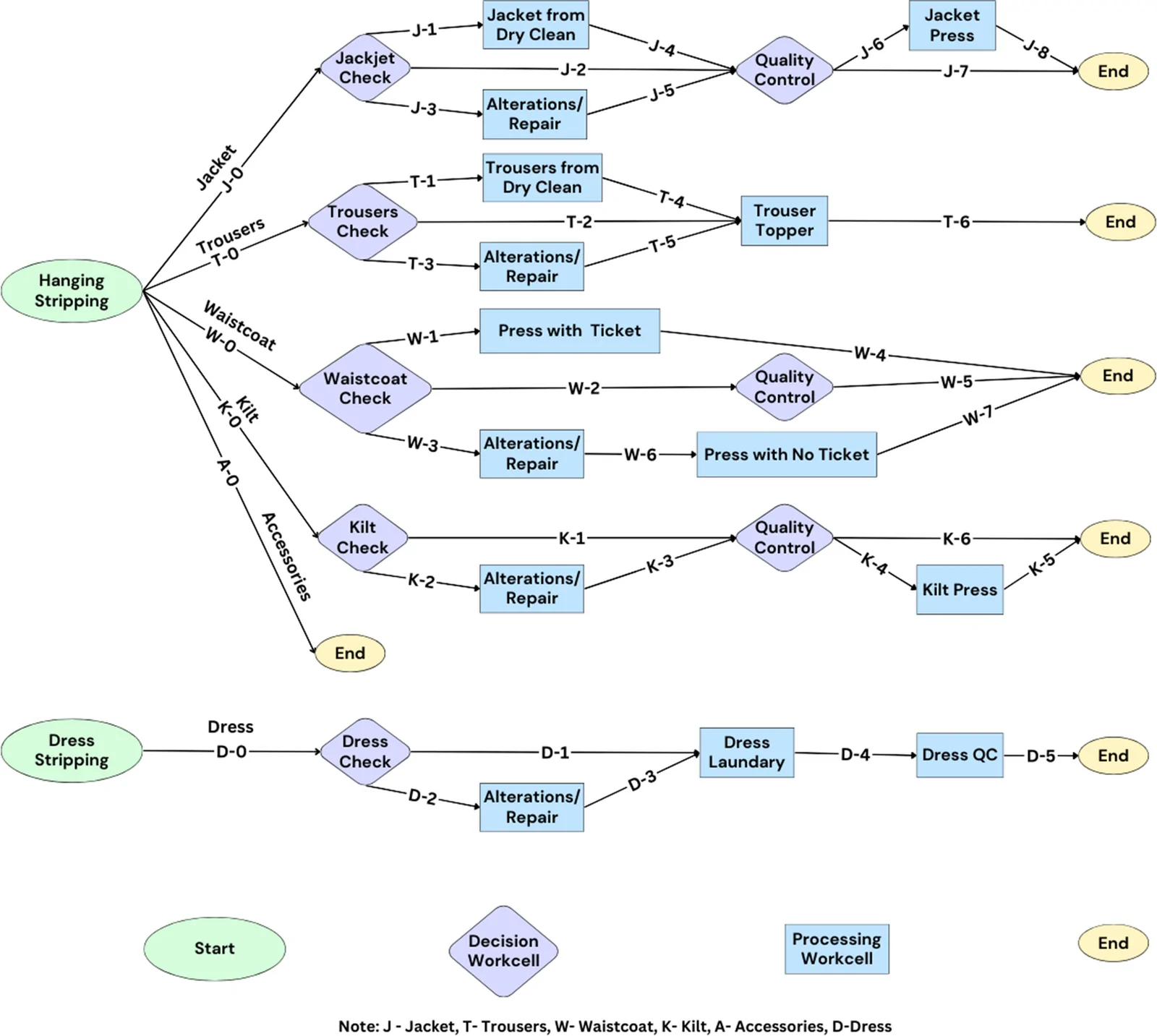
Flow routes of various garments in the network

The garment processing procedure starts with retrieving rented garments from the customers. The unboxing process of each garment is carried out, and subsequent processes are followed to make the garment ready for the customer to rent it again. The detailed process flow diagram of the different garment types (Jacket, Trousers, Waistcoat, Kilt, and Dress) processing is as shown in Fig. 1.

Every individual garment is scanned as per the predefined “Barcodes” assigned to each item of every garment type. As shown in Fig. 1, the process starts at the stripping workcell (Hanging Stripping and Dress Stripping), where various garments are sorted (using barcode) according to garment type, and “hanging and stripping” of each garment is completed. Then, the sorted garment, as per the garment types, is transferred to the subsequent workcell as per the transfer probabilities defined in Table 2.

**Table 2:** Probabilities of garment flow from the present workcell to the next workcell

Jacket							
R	From	To	P	R	From	To	P
J-0	Hanging Stripping	Jacket Check	0.22	J-5	Alterations/Repairs	Quality Control	1.00
J-1	Jacket Check	Jacket from Dry Clean	0.30	J-6	Quality Control	Jacket Press	0.25
J-2	Jacket Check	Quality Control	0.65	J-7	Quality Control	End	0.75
J-3	Jacket Check	Alterations/Repairs	0.05	J-8	Jacket Press	End	1.00
J-4	Jacket from Dry Clean	Quality Control	1.00				
Trousers							
R	From	To	P	R	From	To	P
T-0	Hanging Stripping	Trousers Check	0.19	T-4	Trousers from Dry Cleaning	Trouser Topper	1.00
T-1	Trousers Check	Trousers from Dry Cleaning	0.40	T-5	Alterations/Repairs	Trouser Topper	1.00
T-2	Trousers Check	Trouser Topper	0.50	T-6	Trouser Topper	End	1.00
T-3	Trousers Check	Alterations/Repairs	0.10				
Waistcoat							
R	From	To	P	R	From	To	P
W-0	Hanging Stripping	Waistcoat Check	0.15	W-4	Press With Ticket	End	1.00
W-1	Waistcoat Check	Press With Ticket	0.40	W-5	Quality Control	End	1.00
W-2	Waistcoat Check	Quality Control	0.50	W-6	Alterations/Repairs	Press No Ticket	1.00
W-3	Waistcoat Check	Alterations/Repairs	0.10	W-7	Press No Ticket	End	1.00
Kilt							
R	From	To	P	R	From	To	P
K-0	Hanging Stripping	Kilt Check	0.035	K-4	Quality Control	Kilt Press	0.20
K-1	Kilt Check	Quality Control	0.8	K-5	Quality Control	End	0.80
K-2	Kilt Check	Alterations/Repairs	0.20	K-6	Kilt Press	End	1.00
K-3	Alterations/Repairs	Quality Control	1.00				
Dress							
R	From	To	P	R	From	To	P
D-0	Dress Stripping	Dress Check	1.00	D-3	Dress Check	Dresses out of laundry	0.85
D-1	Alterations/Repairs	Dresses out of laundry	1.00	D-4	Dresses out of laundry	Dress QC	1.00
D-2	Dress Check	Alterations/Repairs	0.15	D-5	Dress QC	End	1.00
Accessories							
R	From	To	P				
A-0	Hanging Stripping	End	40.5				

*R* Route, *P* Probability

To understand the scenario, consider the example as follows: (i) the total number of garments that are scanned at the workcell Hanging Stripping, out of which 22% of the garments are of garment type “Jacket”, which transfer from workcell “Hanging Stripping” to the workcell Jacket Check. (ii) Then, the total number of garments that reach to the workcell Jacket Check, after processing at Jacket Check, 30% of the total garment (Jacket) at Jacket Check is transfer to the workcell Jacket from Dry Clean, 65% of the total garment (Jacket) at Jacket Check is transfer to the workcell Quality Control, 5% of the total garment (Jacket) at Jacket Check is transfer to the workcell Alterations/Repairs. (iii) Next, the total garment that reaches the workcell Jacket from Dry Clean and Alterations/Repairs, after processing the individual garment, 100% of the garment from both the workcell is then transferred to the workcell Quality Control. (iv) The total number of garments that reach the workcell Quality Control, after processing, 25% of the total garments are transferred to the workcell Jacket Press, and 75% of the total garments reach “End” for storage. (v) Finally, 100% of the garments that reach the workcell Jacket Press, after processing, reach “End” for storage.

The route of flow sequence of each scanned barcode garment is not predefined. The allocation of the next workcell is dependent on the garment quality check performed at the decision point workcell (Jacket Check and Quality Control) in the case of the Jacket. From where the route decision is carried out for the subsequent movement of the garment to the next workcell. This scenario suggests the complexity involved in the whole network system, as an individual garment may take different routes. The same probability distribution from one workcell to the next workcell with decision workcells and processing workcells involved in the route network can be applied to other garment types (Trousers, Waistcoat, Kilt, and Dress), considering the Fig. 1 and Table 2.

The transit time for different routes of each garment type is as defined in Table 3. To understand the given transit time for each garment type route flow, consider an example, Let for route “J-0”, transit time: (0, 3, 50), (3, 6, 30), (6, 20, 10), (20, 40, 5), (40, 50, 5) means the total garment (jacket) which transfer from the workcell Hanging Stripping to the workcell Jacket Check, form that 50% garment take 0 to 3 h, 30% garment take 3 to 6 h, 10% garment take 6 to 20 h, 5% garment take 20 to 40 h, and 5% garment take 40 to 50 h to transfer. In the same way, for each route, understand the transit time data given in the Table 3. The Table 3 data show the high variability of transit time to transfer a garment from one workcell to another. This variability of transit time is highly dependent on the priority of one garment type over others. The priority is dependent on the customer demand, seasonal effect, and resource utilization.

**Table 3:** The transit time for different routes of each garment type

Jacket			
Route	From	To	Transit time
J-0	Hanging Stripping	Jacket Check	(0, 3, 50), (3, 6, 30), (6, 20, 10), (20, 40, 5), (40, 50, 5)
J-1	Jacket Check	Jacket from Dry Clean	(0, 0.5, 25), (0.5, 1, 15), (1, 1.5, 30), (1.5, 2, 20), (2, 2.5, 10)
J-2	Jacket Check	Quality Control	(0, 0.5, 25), (0.5, 1, 30), (1, 1.5, 15), (1.5, 2, 20), (2, 2.5, 10)
J-3	Jacket Check	Alterations/Repairs	(1, 1.5, 40), (1.5, 3, 15), (3, 4.5, 25), (4.5, 6, 20)
J-4	Jacket from Dry Clean	Quality Control	(3, 4, 40), (4, 5, 35), (5, 6, 25)
J-5	Alterations/Repairs	Quality Control	(1, 1.5, 45), (1.5, 2, 25), (2, 2.5, 30)
J-6	Quality Control	Jacket Press	(0, 0.5, 25), (0.5, 1, 15), (1, 1.5, 30), (1.5, 2, 20), (2, 2.5, 10)
J-7	Quality Control	End	(0, 0, 100)
J-8	Jacket Press	End	(0, 0, 100)
Trousers			
Route	From	To	Transit time
T-0	Hanging Stripping	Trousers Check	(0, 0.5, 40), (0.5, 5, 35), (5, 15, 15), (15, 40, 10)
T-1	Trousers Check	Trousers from Dry Cleaning	(0, 0.5, 25), (0.5, 1, 30), (1, 1.5, 15), (1.5, 2, 20), (2, 2.5, 10)
T-2	Trousers Check	Trouser Topper	(0.5, 1, 45), (1, 1.5, 25), (1.5, 2, 30)
T-3	Trousers Check	Alterations/Repairs	(1, 1.5, 40), (1.5, 3, 25), (3, 4.5, 15), (4.5, 6, 20)
T-4	Trousers from Dry Cleaning	Trouser Topper	(3, 4, 45), (4, 6, 25), (6, 8, 30)
T-5	Alterations/Repairs	Trouser Topper	(1, 1.5, 45), (1.5, 2, 25), (2, 2.5, 30)
T-6	Trouser Topper	End	(0, 0, 100)
Waistcoat			
Route	From	To	Transit time
W-0	Hanging Stripping	Waistcoat Check	(0, 0.5, 35), (0.5, 3, 30), (3, 10, 10), (10, 25, 25)
W-1	Waistcoat Check	Press With Ticket	(1, 3, 35), (3, 4.5, 40), (4.5, 6, 25)
W-2	Waistcoat Check	Quality Control	(1, 3, 45), (3, 4, 25), (4, 5.5, 30)
W-3	Waistcoat Check	Alterations/Repairs	(1, 1.5, 25), (1.5, 3, 15), (3, 4.5, 40), (4.5, 6, 20)
W-4	Press With Ticket	End	(0, 0, 100)
W-5	Quality Control	End	(0, 0, 100)
W-6	Alterations/Repairs	Press No Ticket	(3, 4, 45), (4, 5, 25), (5, 5.5, 30)
W-7	Press No Ticket	End	(0, 0, 100)
Kilt			
Route	From	To	Transit time
K-0	Hanging Stripping	Kilt Check	(0, 3, 50), (3, 6, 30), (6, 20, 10), (20, 30, 5), (30, 50, 5)
K-1	Kilt Check	Quality Control	(1, 3, 55), (3, 8, 30), (8, 10, 15)
K-2	Kilt Check	Alterations/Repairs	(1, 2, 45), (2, 3, 25), (3, 6, 30)
K-3	Alterations/Repairs	Quality Control	(1, 1.5, 45), (1.5, 2, 25), (2, 2.5, 30)
K-4	Quality Control	Kilt Press	(1, 2.5, 55), (2.5, 5, 35), (5, 6, 10)
K-5	Quality Control	End	(0, 0, 100)
K-6	Kilt Press	End	(0, 0, 100)
Dress			
Route	From	To	Transit time
D-0	Dress Stripping	Dress Check	(0, 0.5, 40), (0.5, 3, 30), (3, 4, 15), (4, 5.5, 10), (5.5, 8, 5)
D-1	Alterations/Repairs	Dresses out of laundry	(1, 1.5, 45), (1.5, 2, 25), (2, 2.5, 30)
D-2	Dress Check	Alterations/Repairs	(0, 0.5, 50), (0.5, 1, 50)
D-3	Dress Check	Dresses out of laundry	(0, 1, 35), (1, 2, 40), (2, 2.5, 25)
D-4	Dresses out of laundry	Dress QC	(0, 0.5, 40), (0.5, 1, 30), (1, 1.5, 20), (1.5, 2, 5), (2, 2.5, 5)
D-5	Dress QC	End	(0, 0, 100)

The total number of garments scanned after unboxing at the workcell Jacket Check and Dress Stripping is considered as the external arrival rate (items/hour) for the whole network system. Table 4 illustrates the number of workers available at each workcell and the processing rate of each worker in items per hour at the workcell.

**Table 4:** The number of workers available at each workcell and the processing rate of each worker in items per hour at the workcell

Workcell	Worker’s processing rate (items/hour)	Number of workers
Hanging Stripping	90	8
Kilt Check	13	2
Jacket Check	23	7
Trousers from Dry Cleaning	14	5
Waistcoat Check	48	2
Trousers Check	19	5
Jacket from Dry Clean	26	2
Dress Check	20	1
Dresses out of laundry	10	4
Alterations/Repairs	8	4
Dress Stripping	12	2
Jacket Press	16	3
Press No Ticket	12	2
Press With Ticket	22	2
Kilt Press	9	1
Quality Control	60	4
Dress QC	20	1
Trouser Topper	26	4

The internal arrival rates (items/hour) for the subsequent workcells depend on the probabilities defined in Table 2 and on the garment quality check performed at the decision point workcell (Fig. 1). The external arrival rate highly depends on the seasonal effect, bank holidays, marriage seasons, and other factors. The M/M/m queueing and probabilistic transition models are extensively used in complex network systems because they effectively accommodate varying transition probabilities, represent the system’s dynamic characteristics, and manage intricate network transitions. The uniform time distribution approach delivers precise performance and lead time estimations, guaranteeing accurate representation of complex systems characterized by fluctuating waiting times and throughput.

## Mathematical model for tradeoff analysis

The garment processing is considered an open Jackson queueing network, as shown in Fig. 1. The open Jackson queue network includes N workcells. The external arrival rate at the workcell * i*, * i* = 1, 2, 3…n is a Poisson distributed with rate $\:{\lambda\:}_{0i}$ (λ ≥ 0, $\:,\forall\:\:i$). A garment completed the service at the workcell * i* transferred to the workcell * j* with a probability $\:{p}_{ij}$ where, 0 ≤ $\:{p}_{ij}$ ≤ 1, $\:\forall\:i$, * j* = 0,…,n and $\:{\sum\:}_{j=0}^{n}{p}_{ij}=1,\:\:\forall\:i$ = 0,…,n. The probability $\:{p}_{i0}\:$represent the fraction of garments leaving the network at the queue. The departure is exponentially distributed. Let $\:{\lambda\:}_{j}$ represent the total arrival rate at the workcell * j*. Then, according to findings by Baulam Rabta [53], the arrival rate (items/hour) at the workcell * j* is expressed as:

1 $$\\\begin{array}{c}\:{\lambda\:}_j={\lambda\:}_{0i}+\sum\:_{i=1\:}^n\\(p_{ij}\times\:{\lambda\:}_i),\:,\forall\:j=1,\dots\:,n\\\end{array}$$

The service rate $\:{\mu\:}_{j}$ (items/hour) of workcell * j*, for the M/M/m queue model with m number of workers working in parallel within the workcell is calculated as:

2 $$\:\\\begin{array}{c}\:{\mu\:}_j=Worker^{'}s\:Processing\:Rate\times\\Number\:of\:workers\:\end{array}$$

Where worker’s processing rate at the specific work cell in items/hour.

The utilization ($\:{\rho\:}_{j})$ of each workcell based on the effective interarrival rate $\:{\lambda\:}_{j}$ and service rate $\:{\mu\:}_{j}$ of workcell is given by:

3 $$\:\left({\rho\:}_{j}\right)=\:\frac{{\lambda\:}_{j}}{{\mu\:}_{j}}$$

For M/M/m queue model, the queue performance is given by:

4 $$\\\begin{array}{c}\:P_q=\\\:\left\{\left[\sum\limits_{j\:=0}^{n-1}\frac{{\rho\:}_j^n}{n!}\right]+\:\frac{{\rho\:}_j^n}{n!(1-\:{\rho\:}_j)}\right\}^{-1}\:\:\:\:,\forall\:j\\\end{array}$$

5 $$\:\\\begin{array}{c}\:L_q=\\\:\frac{{{\lambda\:}_j{\mu\:}_j\left({\rho\:}_j\right)}^n}{\left(n-1\right)!\left({\mu\:}_j-{\lambda\:}_j\right)}\times\\\:P_q\:\:\:,\forall\:j\end{array}$$

6 $$\:L_j=\:L_q+{\rho\:}_{j\:\:\:\:\:\:\:},\:\forall\:j$$

7 $$\:W_q=\:\:\left({\rho\:}_j-1\right)\frac1{{\mu\:}_i}L_{j\:\:\:\:\:\:\:},\:\forall\:j$$

Where:$\:{P}_{q}$ is the probability of no garment in the queue network, $\:{L}_{q}$ is the probability of items waiting in the queue network at each workcell, $\:{L}_{j}$ is the number of garments waiting in the queue (items/hour), $\:{W}_{q}$ is the waiting time in the queue network (hours) of individual garment.

The service time for the individual garment to be processed at each workcell is given by:

8 $$\:Service\:Time=\frac{1}{{\mu\:}_{i}},\:\forall\:\mathrm{j}$$

Each route of garment transit comprises a defined time probability distribution, and the transit time between two workcells (* i* and * j*) is obtained from an individual uniform distribution as given below:

9 $$\:{T}_{ij}\:\sim\:{D}_{u}\:({X}_{a},{X}_{b}),$$

where$$\:\sum\:_{\mathrm{a}}{\mathrm{P}}_{\mathrm{a}}\:=1$$, 

Where, $\:{D}_{u}\:({X}_{a},{X}_{b})$: For [$\:{X}_{a},{X}_{b}$] having a uniform distribution.

*P*_*a*_ :Probability of assigning the k-interval.

The total lead time for each garment entering the network is determined by the probability of workcell allocation and transit time distribution, terminating it by storing the garment at the end is given by:

10 $$\\\begin{array}{c}\:Total\:Lead\:Time\:\left(Product\right)Lp=\\\sum\limits_j(\:T_{ij}\:{+\:W}_{\left(q\right)}+\:\frac1{{\mu\:}_j})\:,\:\forall\:j\\\end{array}\:\:$$

The total Throughput of individual garment type is defined as the total number of garments reaching “End” in the predefined time, and is given by:

11 $$\\\begin{array}{c}\:Throughput\:\theta\:=\\\sum\limits_jmin({\mu\:}_j\times\:(1-{\rho\:}_j)\:L_p),\:\forall\:j\end{array}$$

Assumptions and limitations:

1. For this model, we assumed a unidirectional flow of the garment, indicating that each garment starts its route from the start point (work cell), then travels to subsequent work cells as per the defined flow probabilities and ultimately reaches the End.
2. All workers have equal processing rates on each workcell.
3. The buffer of waiting at each workcell is infinite.
4. Each worker is flexible to work on any workcell.
5. Worker allocation is fixed throughout the working hours.
6. Workers can work in parallel in a single workcell on different garments.
7. The total number of workers in the whole network is fixed.

### Performance measures evaluation algorithm

This section briefly outlines the decomposition approach for measuring metrics for the performance of an open Jackson queueing network. In a dynamic network with many nodes and linked workcells, the decomposition approach refers to splitting the complex network into independent workcells, considering each workcell as a unique queue with an effective inter-arrival rate derived from the network’s probability distribution. It is based on the combined probability distribution of the state of a network and is characterized as a combination of independent node probabilities [65, 66]. It expresses the queue independence assumption between the nodes. However, assuming upstream congestion while neglecting dynamics and transient behavior may constrain performance in a highly utilized network [22]. It is efficient in memoryless operations with large buffers and a little real-time input from associated nodes [67, 68]. Chandra and Jain [69] examined the interdependency value of decomposition for rapid decision support in complex systems, while Lim and Singh [70] acknowledged that their assumptions limit fidelity under extreme variability or tightly coupled workflows in the decomposition model. The modular nature of garment reprocessing, with all workcells having independent resources and probabilistic routes for each garment, promotes the application of decomposition within the open Jackson queueing network system. The approximation of the predicted lead process time and throughput of each garment type can be validated using the interview and past processing data. The decomposition approach primarily includes three steps. The first step involves the decomposition of the complex network system into an independent system. In the second step, performance measure (utilization) is evaluated at the individual workcell, and in step three, the findings of step 2 are merged to evaluate performance metrics for the entire network.

The decomposition technique is an approximation process acknowledged in the literature for its efficiency. The level of accuracy is acceptable in various real-world situations. We investigate a single day shift in which all workers operate for 8 h to measure the utilization of each workcell, throughput, and lead time for each garment type.

**Figure:**
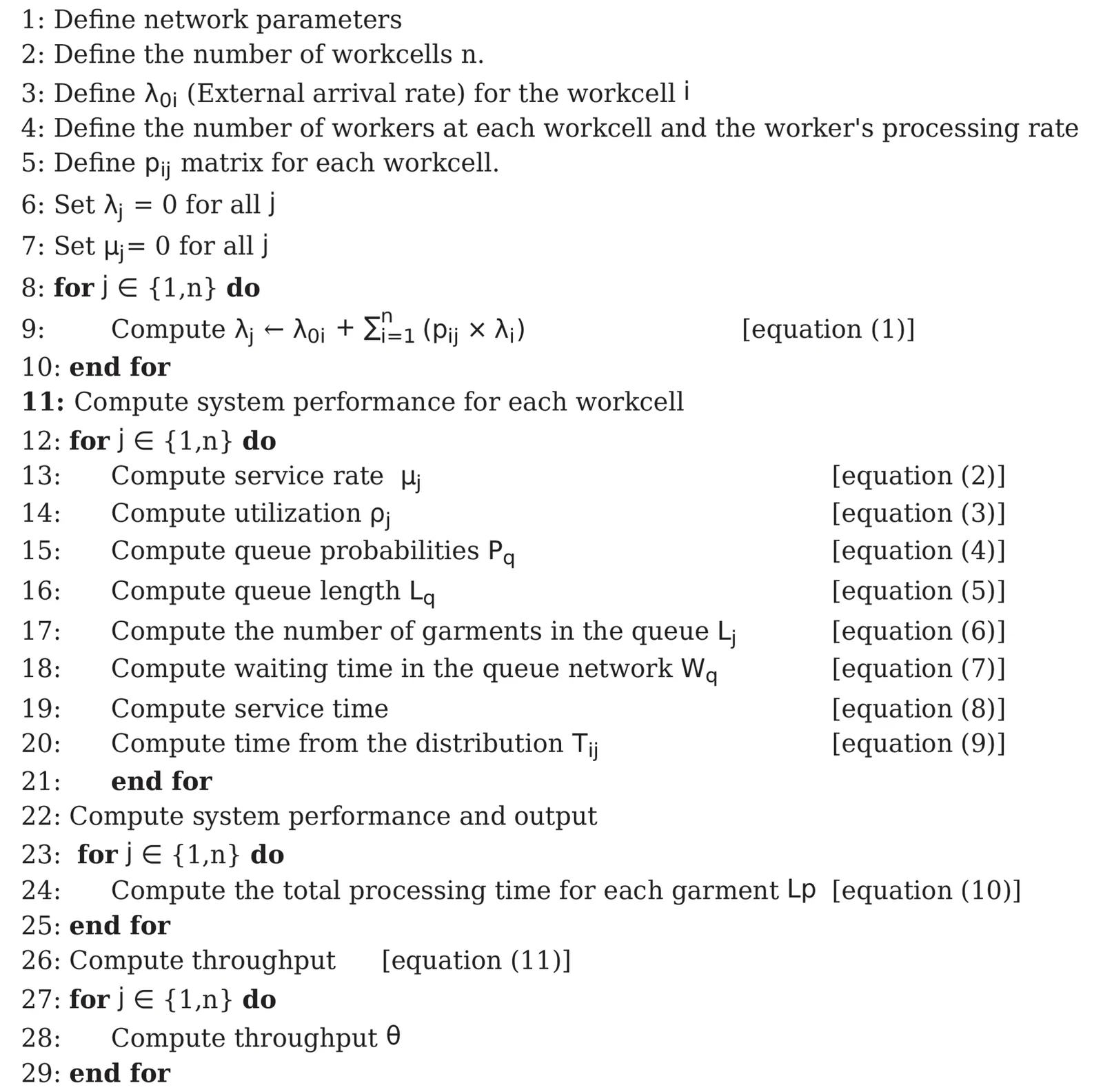
Algorithm Pseudo code of the proposed decomposition of the open Jackson queueing network model

## Results and discussions

**Fig. 2:**
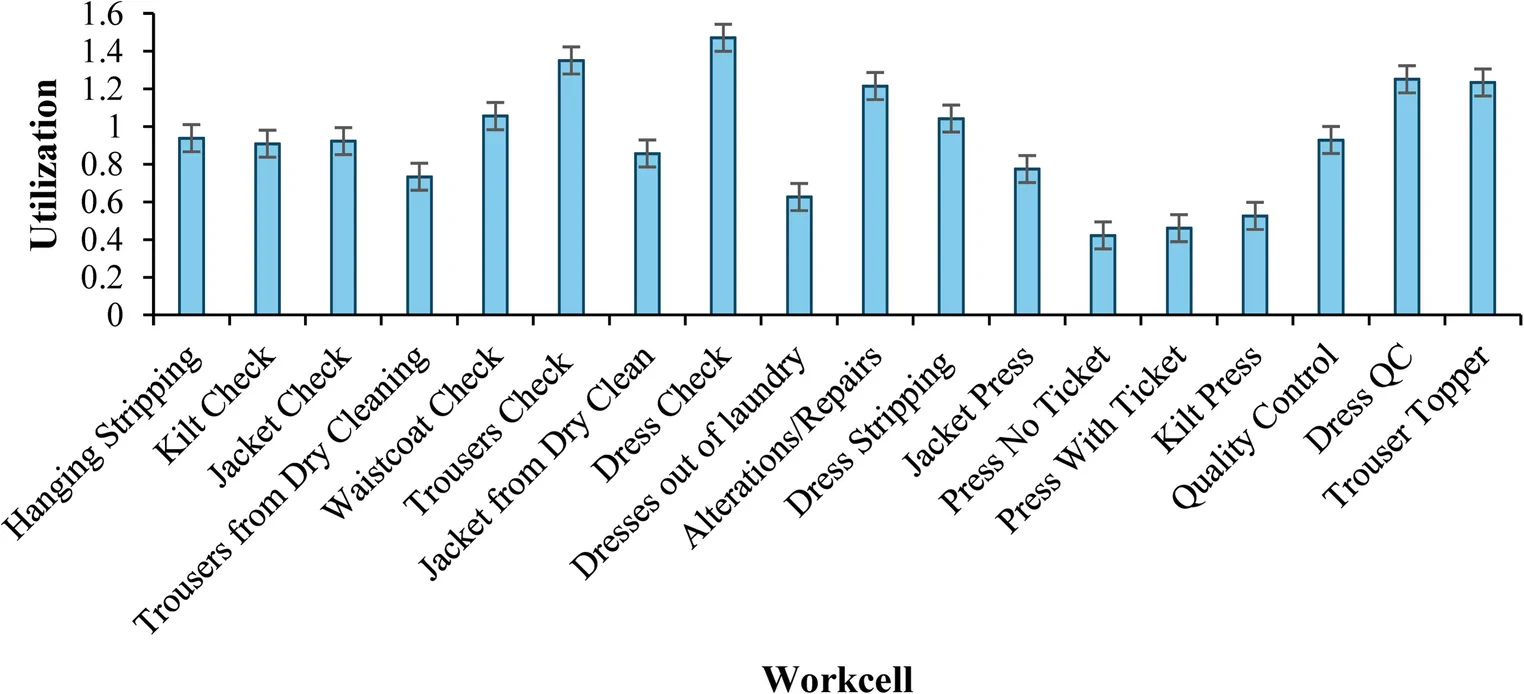
Utilization of each workcell in the present (initial) scenario

To understand the effect of arrival rate and worker’s processing rate in the complex network system of the garment processing network as described in Sect. 4 above, the first step is to understand the resource utilization of the present network layout. The utilization of the decomposition technique provides details of the resource utilization, Lead time, and throughput of each garment type. Figure 2 shows the present scenario of the workcell utilization, which shows that the workcell Hanging Stripping, Kilt Check, Jacket Check, Waistcoat Check, Dress Stripping, and Quality Control have a utilization rate of approximately 1. The workcell Trousers from Dry Cleaning, dresses out of laundry, Jacket Press, Press No Ticket, Press with Ticket, and Kilt Press are between 0.4 and 0.8, means underutilization, while the workcell Trousers Check, Dress Check, Alterations/Repairs, Dress QC, and Trouser Topper is over utilized with utilization rate from 1 to 1.5. The computed utilization rate varies depending on the dynamic characteristics of the flow network, governed by probability distribution, decision point work cell, priority, and transit time.

**Fig. 3:**
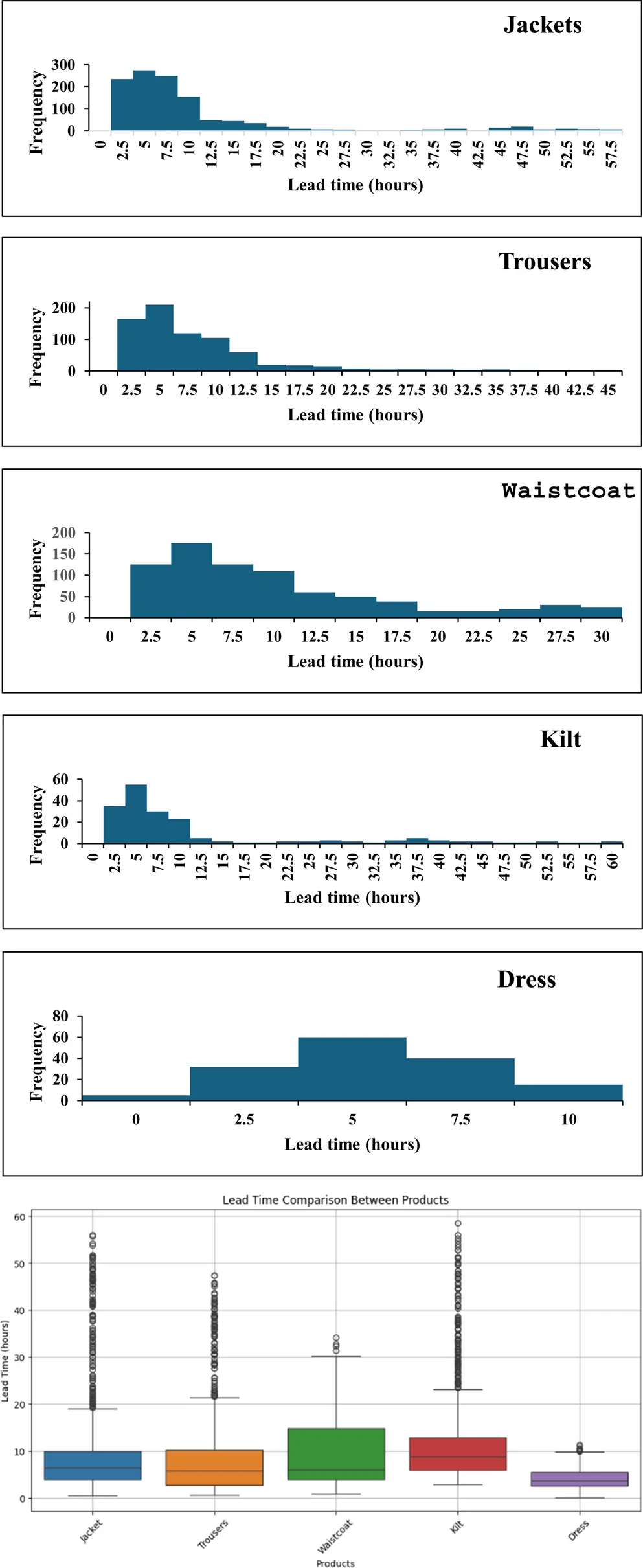
Histogram plot of the lead time distribution and comparison box plot of lead time for the garment types

Figure 3 displays histogram plots of the lead time distribution for the various garment types. The lead time distribution for jackets ranges from 2 h to 50 h. For trousers, 1 to 40 h, for waistcoat, 1 to 30 h, for kilt, 1 to 60 h, and for dress, 1 to 10 h. Each garment’s lead time range depends on the probability distribution from the check workcell, transit time distribution, and the workcell utilization variations. The boxplot compares the interquartile range (IQR: 25th to 75th percentile) of lead time in the hours of each garment type. In the boxplot, the Whiskers range up to 1.5 times IQR or the most extreme data points in this range. The small circles outside the whiskers are considered outliers. All garment types except dress have a broader distribution of lead time and many outliers. The IQR range for each garment type in hours is as follows: Jacket (3.91, 10.03), trousers (2.78, 10.20), waistcoat (4.01, 14.99), kilt (5.96, 12.870, dress (2.53, 4.58). The horizontal line inside the box represents the median lead time for each garment type. The median lead time in hours is just over 8 h for kilts, but under 6.5 h for jackets, trousers, and waistcoat (6.16, 6.14, and 5.64 h, respectively). However, the dress has a narrower distribution and has a lead time of less than 4 h. For all garment types, the median line in the boxplot (IQR) is “Right- Skewed,” suggesting the median time is approaching the lower limit of the box plot.

### Tradeoff curve analysis

In manufacturing systems, arrival rates are critical because they are important in determining system efficiency and throughput. The arrival rate might influence production capacity, such as system stability, processing delays, or resource limitations. They directly impact lead times, which are critical to meeting production schedules and maintaining customer demand. Insights into the relationship between lead times and arrival rates can help process managers with resource allocation and production schedules. The arrival rate in the rental business model is significantly affected by the seasonal effects, wedding seasons, bank holidays, and Christmas holidays, etc. In the first quarter of the year after Christmas, customer demand is found to be low, then in the second quarter, due to bank holidays, customer rental demand slightly increases. In the third quarter, during the summer and the school holiday, the customer’s garments are at their peak. Finally, in the last quarter of the year, demand initially decreases, but at the end of year, the demand increases again due to the Christmas celebration. Hence, the tradeoff curves of sensitivity analysis of arrival rate variations of the different garment types are helpful to the process manager to take strategic actions in garment processing to prepare the garment for ready supply to the customer on demand in both low and peak demand of garments. In this work, to study the sensitivity analysis, the external arrival rate is reduced to 20% of the current external arrival rate and increased up to 50% to see the effect on lead time and throughput. The data on the external arrival rate is as shown in Table 5.

**Table 5:** Variations in external arrival rates

External Arrival Rate (Items/hour)				
0.8 Arrival rate	0.9 Arrival Rate	1 Arrival Rate	1.25 Arrival Rate	1.5 Arrival Rate
540	608	675	845	1012

#### Effect of external arrival rate variations on the lead time of garment types

**Fig. 4:**
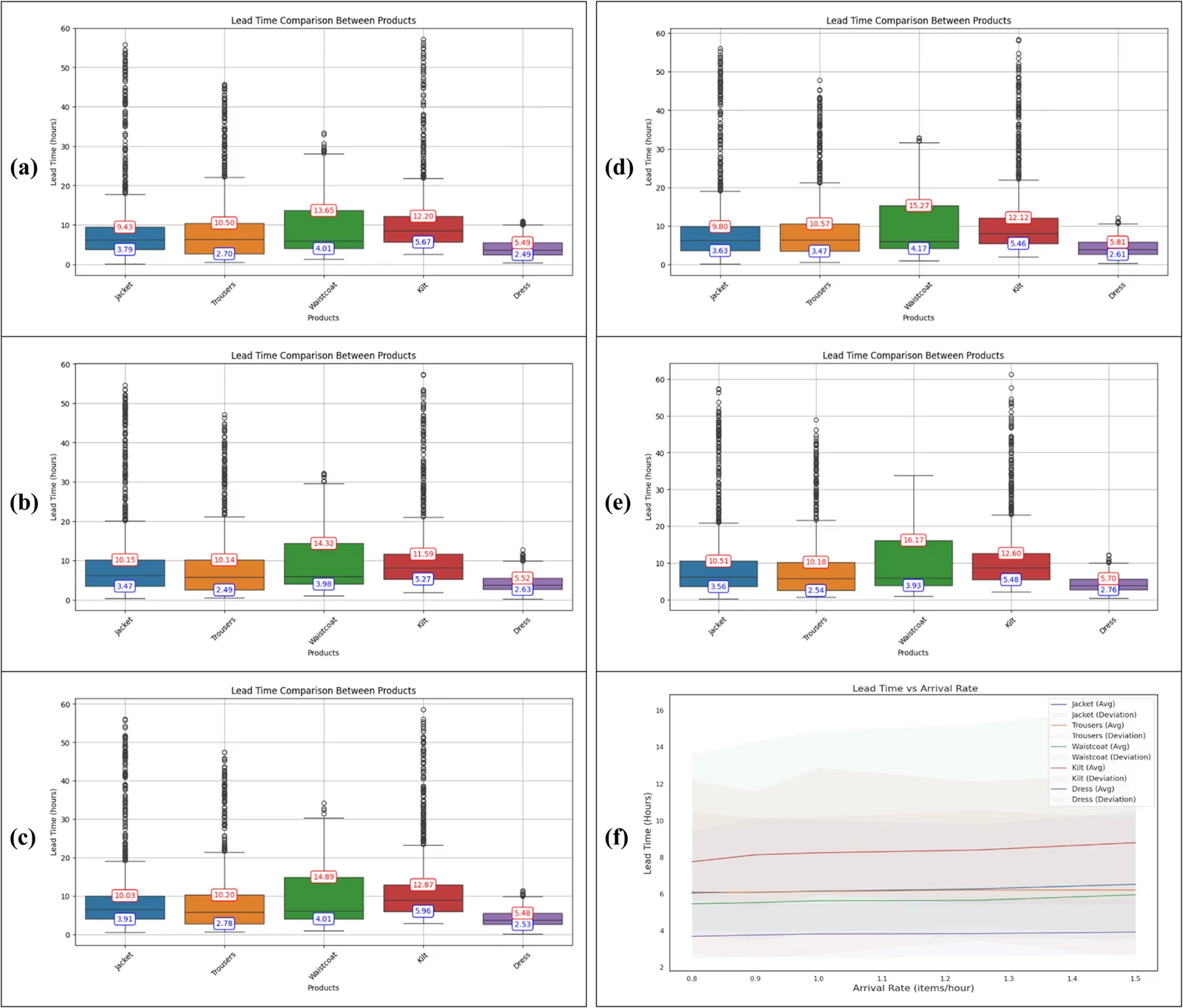
Box plots of lead times comparisons of different garment types: (**a**) at 0.8 arrival rate, (**b**) at 0.9 arrival rate, (**c**) at 1 arrival rate, (**d**) at 1.25 arrival rate, and (**e**) at 1.5 arrival rate. (**f**) tradeoff curves of different garment types for lead time vs. arrival rate

The tradeoff curves are useful to analyse the lead time change of each garment in the network due to the effect of variation in arrival rate reduced to 0.8 (−20%) and increased to 1.5 (50%) of the current arrival rate. From Fig. 4 (a), the median lead time for jacket is observed to increase marginally with an increase in arrival rate, with a peak of 10.51 h at a 1.5 arrival rate. The median lead time is 6.09 h at 0.8 arrival time, gradually increasing to 6.53 h at 1.5 arrival rate. Lead times exhibit a slight increase at higher arrival rates, while the variability (maximum and minimum) demonstrates a marginal increase, indicating a potential for increased delays over time. For trousers (Fig. 4 (b)), median lead time steadily increases between 6.05 and 6.22 h. The lower IQR shows notable deviations, increasing from 2.7 h (at 0.8) to 3.47 h (at 1.25), prior to falling slightly. Lead time of trousers exhibits considerable consistency across diverse arrival rates, rendering it less susceptible to fluctuations in external arrival rates. The waistcoat shows a noticeable increase in upper IQR lead time, from 13.65 h (0.8) to 16.17 h (1.5), as shown in Fig. 4 (c). The mean lead time increases progressively from 5.47 to 5.96 h. The IQR span is broader, representing higher variability in lead time compared to other garment types.

The mean lead time of a kilt (Fig. 4 (d)) linearly increased from 7.76 to 8.8 h (13% rise) with the increase in arrival rate. Rising arrival rates lead to consistently increasing mean and lower IQR lead times, while having little effect on the maximum time spent. The mean lead time steadily increases from 3.7 to 3.93 h for dress, as shown in Fig. 4 (e). The same trend was observed for upper IQR (5.49 to 5.8 h) and low IQR (2.49 to 2.76 h), with variation in arrival rate from 0.8 to 1.5 times the arrival rate. Increased arrival rates, as can be seen in Fig. 4 (f) cause substantial variations in the average and maximum lead time of waistcoats and kilts, appearing less sensitive to trousers and dresses. The lead time variation for jackets and trousers is linear and increases slightly with varying arrival rates. At high arrival rates, waistcoats and kilts are most affected, while at low arrival rates, all garments tolerate little deviations in the lead time.

#### Effect of external arrival rate variations on the throughput of garment types

**Fig. 5:**
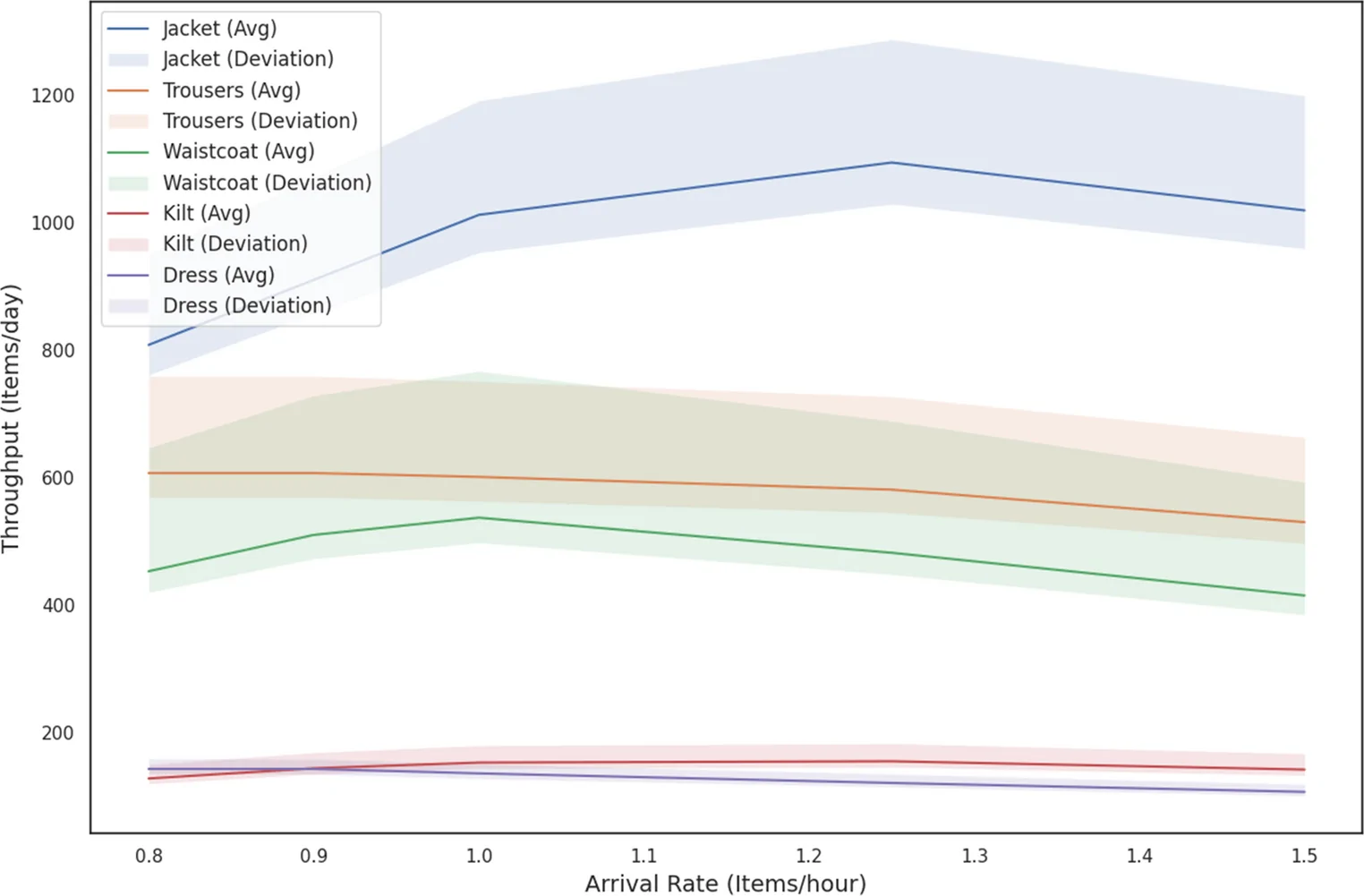
Tradeoff curves of different garment types for arrival rate vs. throughput

Figure 5 shows the trend in average throughput with maximum and minimum corresponding deviation throughput values corresponding to the variation in the arrival rate from 0.8 to 1.5 times the current arrival rate. For Jacket, at an arrival rate of 1, the average throughput is 1013 items per day. When the arrival rate decreases to 0.9 and 0.8, the throughput further declines to 911 and 809 items per day, respectively. As the arrival rate scaled to 1.25 and 1.5, the throughput increased to 1095 items per day at 1.25 but declined to 1020 items per day at 1.5. With an arrival rate of 1, the average throughput of trousers is 602 items daily.

The rates increased to 608 items per day at 0.9 and remained constant despite a subsequent reduction in the arrival rate to 0.8. Conversely, an increase in the arrival rate to 1.25 and 1.5 results in a further reduction in the average throughput to 582 and 531 items per day, respectively. The average throughput of the waistcoat has a positive trend with the arrival rate, increasing from 454, 511, and 538 items per day for arrival rates of 0.8, 0.9, and 1, respectively. As the arrival rate increased to 1.25 and 1.5, the average throughput exhibited a declining trend, decreasing to 484 and 416 items per day, respectively. Identical to the waistcoat, the kilt experienced an uptrend between arrival rate and throughput, increasing from 129 items per day to 156 items per day at a 1.25 arrival rate; however, it then decreased to 415 items per day at a 1.5 arrival rate. The dress exhibits an average throughput of 137 items per day at an arrival rate of 1. At an arrival rate of 0.9, the average throughput increases to 144 items per day, and it remains consistent at an arrival rate of 0.8 as well. Moreover, a rise in the arrival rate from 1.25 to 1.5 results in a decrease in average throughput to 122 and 108 items per day.

The tradeoff curves for multiple garment types in Fig. 5 illustrate how the arrival rate impacted each garment throughput. As the arrival rate rises from 0.8 to 0.9, the throughput of jackets, waistcoats, and kilts increases, but the throughput of trousers and dresses declines. At an arrival rate of 1, the waistcoat and kilt exhibited their greatest average throughput across all ranges of arrival rates. The jacket exhibited enhanced throughput, but the throughput of pants and dresses decreased. At an arrival rate of 1.25, the jacket exhibited its peak average throughput across all arrival rate ranges. All other garments had a reduced average throughput in comparison to the first arrival rate. An additional rise in the arrival rate to 1.5 resulted in a decline in the average throughput of all garments. Consequently, the tradeoff analysis offers critical insights on the selection of arrival rates to achieve the necessary throughput for a certain garment type.

**Fig. 6:**
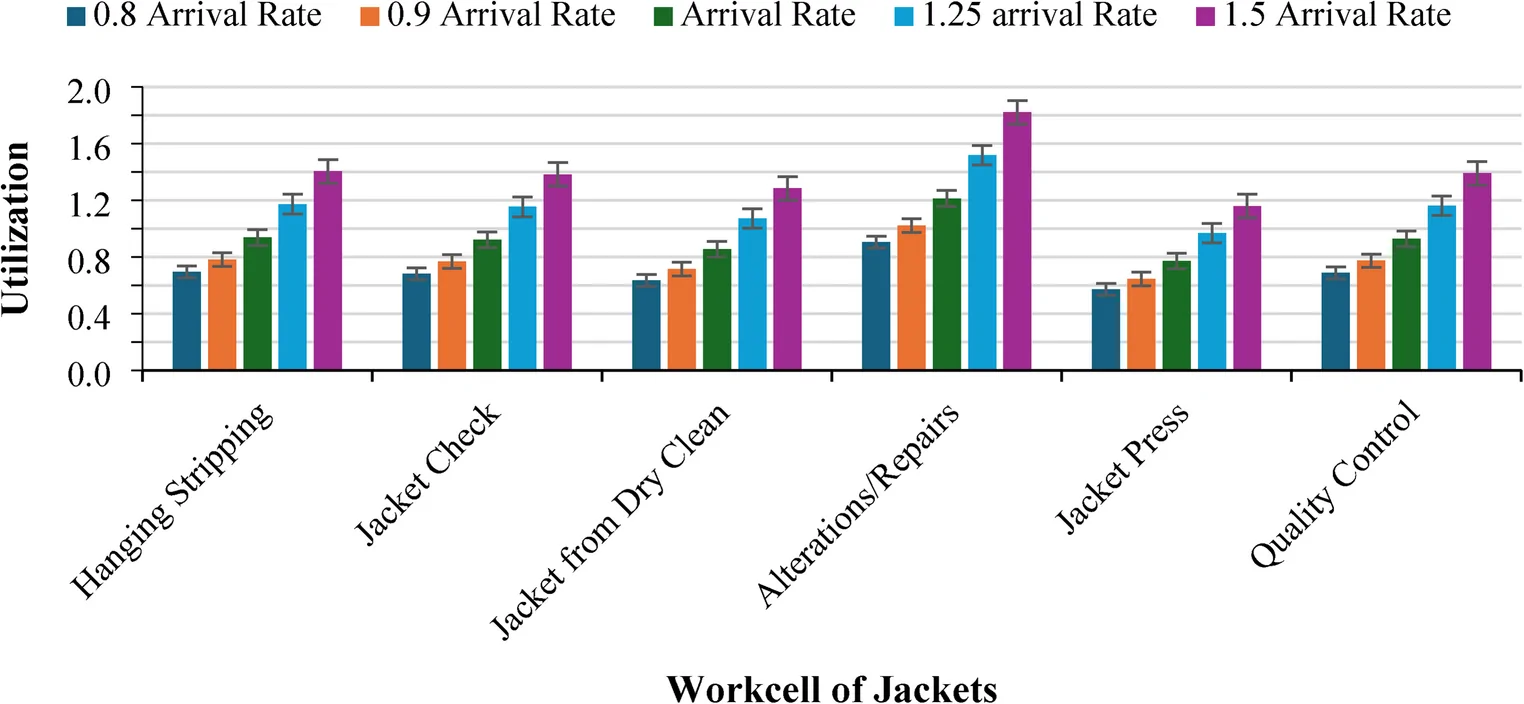
Utilization of the workcells in Jacket processing in varying from 0.8 arrival rate to 1.25 arrival rate

The decomposed workcell utilization analysis of the Jacket, as shown in Fig. 6, reveals that with an arrival rate of 0.8 at hanging stripping, the utilization rate is 0.7. This signifies that the present arrival rate is insufficient to fully exploit the capability of the workcell, with the subsequent workcells demonstrating utilization rates below 1, as seen in Fig. 6. Hence, inadequate network usage resulting from a decline in arrival rate adversely impacted throughput, keeping an average of 809 items per day and a minimum of items waiting at the workcell, resulting in minimal lead time. An identical pattern of the workcell use (ranging from 0.6 to 0.8) is observed for arrival rates of 0.9 and 1, demonstrating a notable increase in throughput to 911 and 1013 items per day, but with a modest increase in median lead time. The further increase in the arrival rate to 1.25 raises the utilization of all workcells from 1.0 to 1.1, leading to an increase in average throughput, but not by the same amount as the 0.9 and 1 arrival rates indicated. The median lead time increased to 6.28 h. Ultimately, as the arrival rate increased to 1.5, all workcells exhibited utilization between 1.4 and 1.7, resulting in a queue of items waiting for processing at each workcell. Consequently, throughput showed a declining trend and decreased to 1020 items per day, and the median lead time went up to 6.53 h. Overall, the jacket exhibited an upward trend in average throughput, increasing from an arrival rate of 0.8 to 1.1. However, a further increase in the arrival rate resulted in a decline in average throughput and an increase in median lead time.

**Fig. 7:**
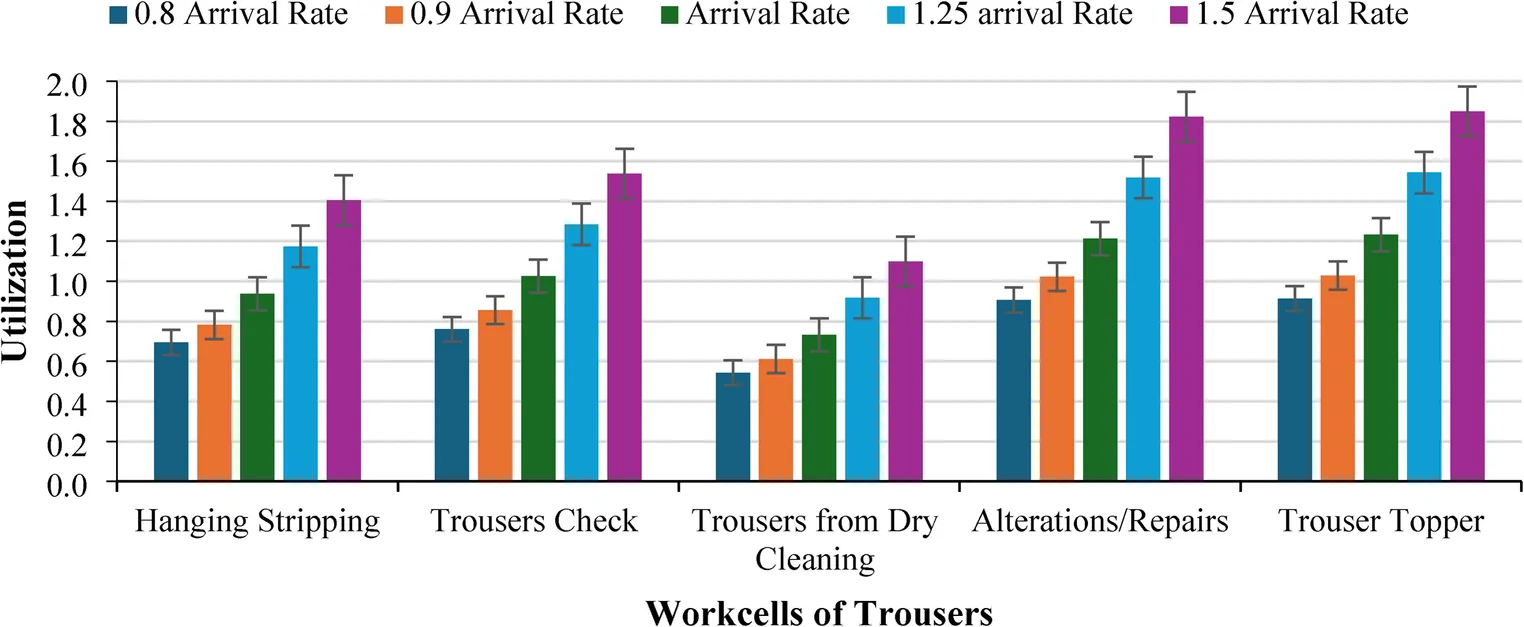
Utilization of the workcells in Trousers processing in varying from 0.8 arrival rate to 1.25 arrival rate

Figure 7 illustrates the segmented utilization of trousers workcells, indicating that at arrival rates of 0.8 and 0.9, the utilization of hanging stripping and trousers check ranges from 0.7 to 0.85, while managing the quantity of items transferred to subsequent workcells maintains a utilization below 1. The average throughput is 540 and 608 items per day, with median lead times of 6.05 h and 6.11 h, respectively. At an arrival rate of 1, as seen in Fig. 7, the utilization for hanging stripping, trouser checks, and trousers from dry cleaning approaches 1, whereas the utilization rates for alteration/repair and trouser toppers range from 1 to 1.2. This results in a slight decrease in the average throughput to 602 items per day with a median time of 6.05 h. Figure 7 illustrates that as the arrival rate increased from 1.25 to 1.5, all workcells exhibit utilization ranging from 1.2 to 1.8, signifying an increase in queue length. This leads to a reduction in average throughput to 582 and 531 items per day, while the median lead time rises to 6.2 h.

**Fig. 8:**
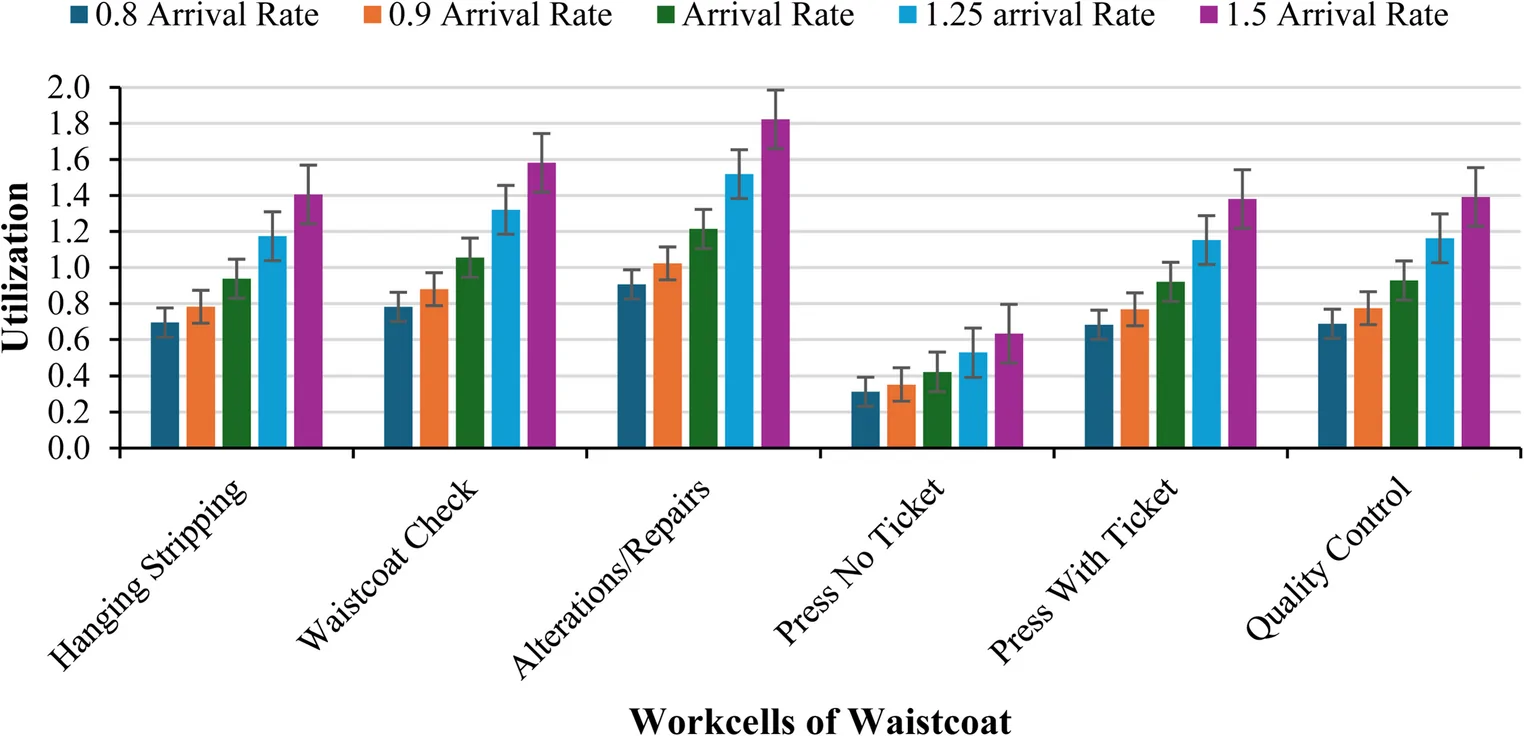
Utilization of the workcells in waistcoat processing in varying from 0.8 arrival rate to 1.25 arrival rate

Referring to Fig. 8, the utilization rate of all workcells for waistcoats at arrival rates of 0.8 and 0.9 is below 1, indicating inadequate resource use; subsequently, the average throughput is 454 and 510 items per day, respectively. As the arrival rate rises to 1, the utilization of workcells ranges from 0.9 to 1.2, leading to an improved average throughput of 538 items per day, with a median duration of 5.64 h. As the arrival rate increases to 1.25 and 1.5, utilization rises to 1.4 and 1.6, resulting in higher waiting times. Therefore, the average throughput decreases to 483 and 416 items per day, with median lead times of 5.66 h and 5.96 h, respectively. The variation in the arrival rate outside a specific range has decreased the average throughput of the garment.

**Fig. 9:**
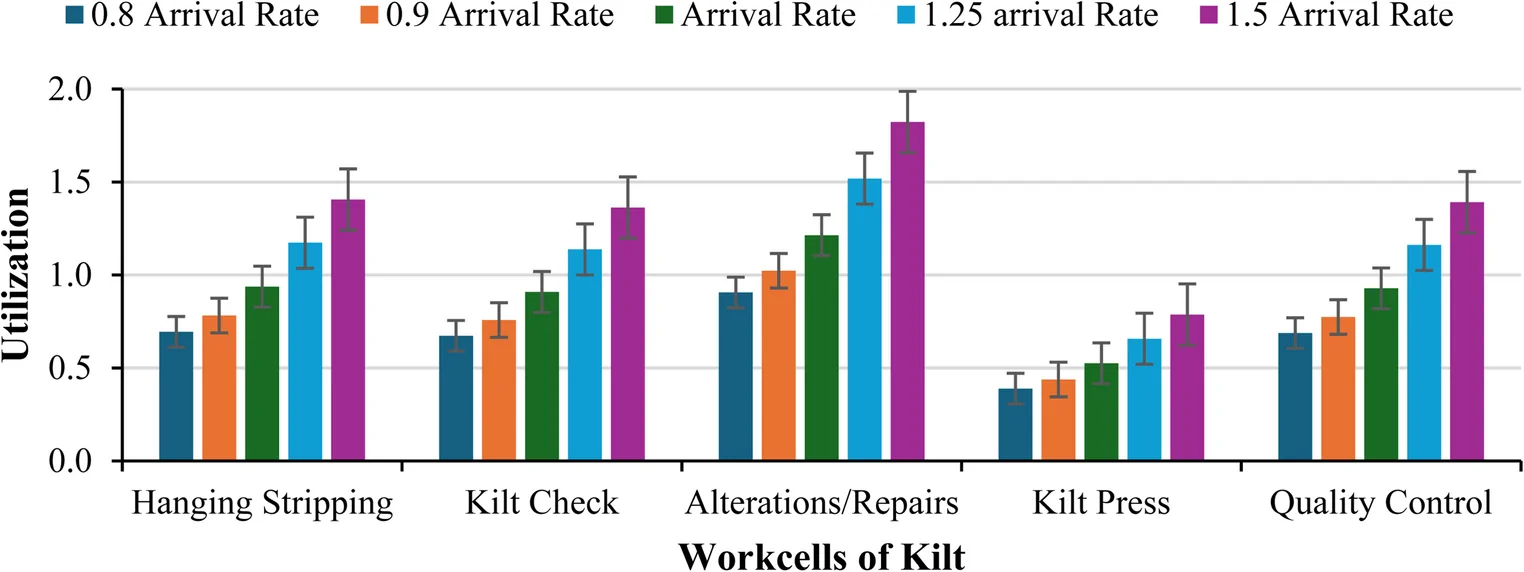
Utilization of workcells in kilt processing in varying from 0.8 arrival rate to 1.25 arrival rate

In reference to the kilt, Fig. 9 illustrates that from 0.8 to 1 arrival rate, the utilization of all workcells fluctuates between 0.6 and 0.8 (except alteration/repair at 1 arrival rate). The reduced utilization rates of hanging stripping and kilt check indicate that less apparel arrives at the system than the network’s actual capacity.

The average throughput is reported at 129 items per day with a 0.8 arrival rate, increasing to 144 items per day at a 0.9 arrival rate, and reaching 154 items per day at a 1 arrival rate, with corresponding median lead times of 7.76 h, 8.14 h, and 8.25 h, respectively. The further increase in the arrival rate to 1.25 and 1.5 results in utilization varying from 1.2 to 1.8, indicating an increase in the number of items waiting for processing at the subsequent workcell. It shows a slight increase in average throughput to 156 items per day at an arrival rate of 1.25, with a median time of 8.4 h. However, this throughput decreases to 143 items per day with a further increase in the arrival rate to 1.5, leading to a median lead time of 8.8 h. In summary, the kilt observed an increasing trend in average throughput with an increase in arrival rate, supported by a steady rise in median lead time. However, further increases in arrival rate resulted in a fall in average throughput with an increase in median lead time.

**Fig. 10:**
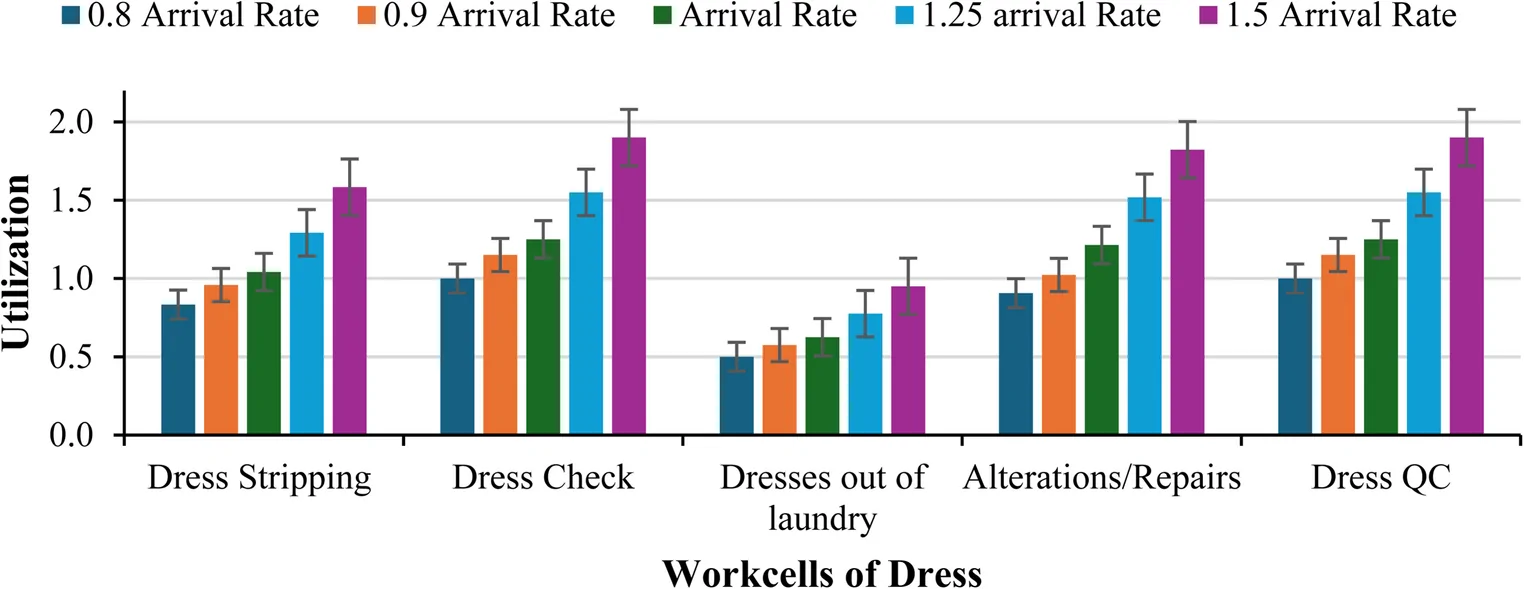
utilization of the workcells in dress processing in varying from 0.8 arrival rate to 1.25 arrival rate

As demonstrated in the graph of the workcell utilization for dress (Fig. 10), for 0.8 and 0.9 arrival rates, all workcells exhibit utilizations below 1 (except dress QC), indicating a consistent average throughput of 144 items per day and a median lead time of 3.7 h. Simultaneously, a consistent rise in arrival rates of 1, 1.25, and 1.5 has led to the workcell utilization rates between 1.2 and 1.8, eventually leading in a decreasing trend for average throughput of 137, 122, and 108 items per day, with median lead times of 3.83 h, 3.85 h, and 3.93 h, respectively. The constantly increasing arrival rates at the workcells associated with dresses gradually decrease average throughput and increase median lead time.

#### Effect of worker’s processing rate variations on the lead time of garment types

The variation in worker’s processing rate substantially affects the efficiency and performance of the production processes. This may result in imbalances, bottlenecks, and resource underutilization, impacting production flow, quality control, and on-time shipment. Implementing standardized procedures and effective training programs is essential for developing methods that reduce their impact and improve productivity. Consequently, the tradeoff among garment types is explored by adjusting the worker’s processing rate, as shown in Table 6 to evaluate its impact on the average throughput and lead time of each garment type.

**Table 6:** Variations in worker’s processing rate and the number of workers assigned to each workcell

Workcells	Worker’s processing rate (Items/hour)					Number of workers
	0.8	0.9	1.0	1.1	1.2	
Hanging Stripping	72	81	90	99	108	8
Kilt Check	10.4	11.7	13	14.3	15.6	2
Jacket Check	18.4	20.7	23	25.3	27.6	7
Trousers from Dry Cleaning	11.2	12.6	14	15.4	16.8	5
Waistcoat Check	38.4	43.2	48	52.8	57.6	2
Trousers Check	20	22.5	25	27.5	30	5
Jacket from Dry Clean	20.8	23.4	26	28.6	31.2	2
Dress Check	16	18	17	22	24	1
Dresses out of laundry	8	9	10	11	12	4
Alterations/Repairs	6.4	7.2	8	8.8	9.6	4
Dress Stripping	9.6	10.8	12	13.2	14.4	2
Jacket Press	12.8	14.4	16	17.6	19.2	3
Press No Ticket	9.6	10.8	12	13.2	14.4	2
Press With Ticket	17.6	19.8	22	24.2	26.4	2
Kilt Press	7.2	8.1	9	9.9	10.8	1
Quality Control	48	54	60	66	72	4
Dress QC	16	18	20	22	24	1
Trouser Topper	20.8	23.4	26	28.6	31.2	4

**Fig. 11:**
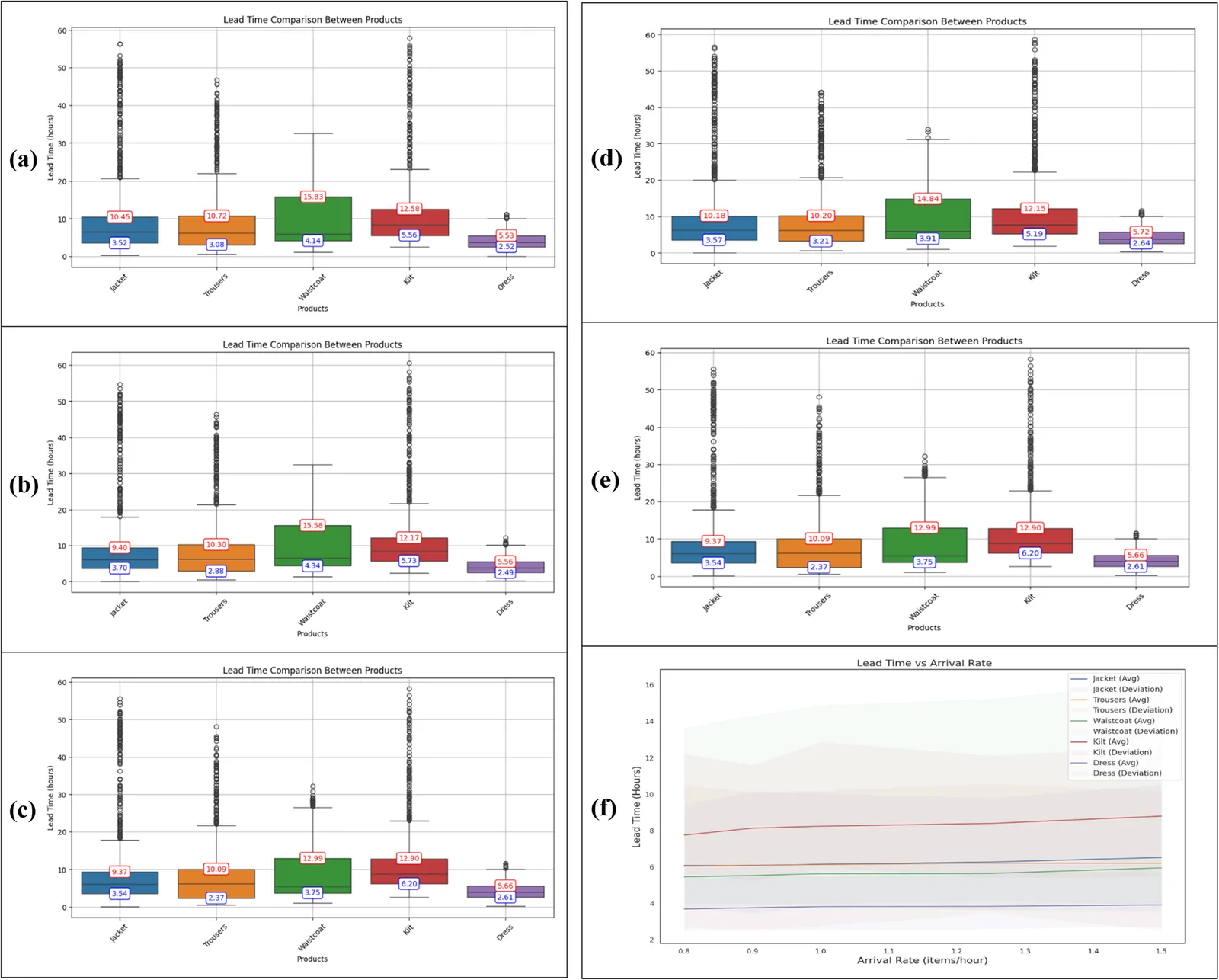
Box plots of lead times comparisons of different garment types : (**a**) at 0.8 worker’s processing rate, (**b**) at 0.9 worker’s processing rate (**c**) at 1 worker’s processing rate, (**d**) at 1.1 worker’s processing rate, and (**e**) at 1.2 worker’s processing rate. (**f**) tradeoff curves of different garment types for worker’s processing rate vs. lead time

The external arrival rate is 675 items per hour at hanging stripping and 25 items per hour at dress stripping. The median lead time for jackets decreased progressively from 6.49 h at a processing rate of 0.8 worker’s processing rate to 5.93 h at a rate of 1.2 worker’s processing rate, as shown in Fig. 11(a). The upper interquartile range of lead time decreased from 10.45 to 9.37 with an increase in the worker’s processing rate, whereas the lower interquartile range of lead time showed slight variation with changes in the worker’s processing rate. The median lead time for trousers (Fig. 11(b)) decreased steadily from 6.35 h at 0.8 worker’s processing rate to 5.86 h at 1.2 worker’s processing rate. The reduced lead time decreased from 3.08 h to 2.37 h. Nevertheless, the top lead time remained mostly stable despite fluctuations in the worker’s processing rates.

The Fig. 11(c) illustrates that the waistcoat demonstrated a significant reduction in upper lead time, decreasing from 15.83 h to 12.99 h at 0.8 to 1.2 worker’s processing rate. The median lead time exhibited modest fluctuations between 5.77 and 5.96 h, however the lower lead time continuously diminished from 5.77 h to 5.96 h. The range of the box plot decreased as the worker’s processing rate increased. The results indicate that increased worker’s processing rate effectively decreases peak times. The median lead time for kilt (Fig. 11(d)) significantly decreased from 8.66 h with 0.8 worker’s processing rate to 7.75 h at 1.2 worker’s processing rates. Both upper lead time (12.58 to 12.9 h) and lower lead time (5.56 to 6.2 h) increase with an increase in the worker’s processing rate. This variation indicates a broader lead time span due to an increase in the workers’ processing pace. The dress, as shown in Fig. 11(e)), exhibited consistency with slight variations in upper lead time (ranging from 5.53 to 5.66 h) and lower lead time (ranging from 2.52 to 2.61 h), while the median lead time appears to decline from 3.95 to 3.53 h as the worker’s processing rate increases. For each garment category (Fig. 11(f)), an increase in the worker’s processing rate positively affects the median lead time, demonstrating enhanced overall efficiency across all garment types. Despite the reduction in median lead time for all garments, certain clothing items (trousers, kilt) exhibit variability in their upper and lower lead times.

#### Effect of worker’s processing rate variations on the throughput of garment types

**Fig. 12:**
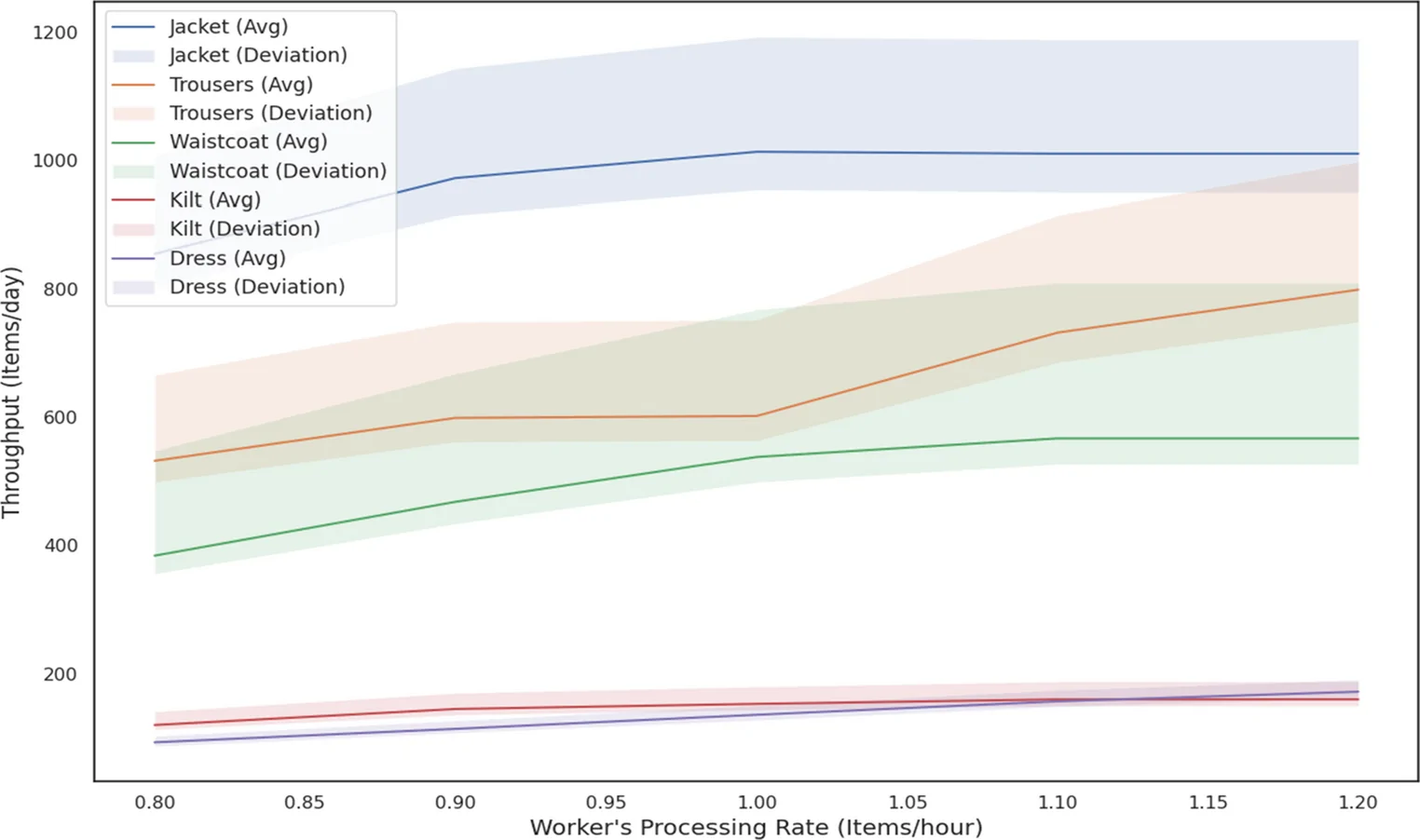
Tradeoff curves of different garment types for worker’s processing rate vs. throughput

Figure 12 shows the trend in average throughput, with maximum and minimum corresponding deviations in throughput values corresponding to the variation in the worker’s processing rate (items per hour) from 0.8 to 1.2 times the worker’s current processing rate (items per hour).

At a worker’s processing rate of 0.8, the average throughput for Jacket is 855 items per day. As the worker’s processing rate increases to 0.9, the average throughput increases to 971 items per day. At a 1 worker’s processing rate worker, it reaches 1,013 items per day. However, with a further increase in the worker’s processing rate to 1.1 and 1.2, the average throughput stabilizes at 1009 items per day. The waistcoat (Fig. 12), similar to the jacket, exhibits an increase in average throughput from 384 to 538 items per day when the worker’s processing rate rises from 0.8 to 1, then stabilizes at 567 items per day at the worker’s processing rate of 1.1 and 1.2. Trousers initially at 0.8 to 1 worker’s processing rate exhibited a gradual rise in average throughput from 532 to 601 pieces per day. Subsequently, with further increments in the worker’s processing rate to 1.1 and 1.2, a substantial increase in average throughput to 798 items per day was observed. Kilt exhibited an upward trend in the relationship between the worker’s processing rate and throughput, rising from 121 items per day to 153 items per day when the worker’s processing rate increased from 0.8 to 1. However, it then steadies at 161 items per day with a further increase in the worker’s processing rate to 1.2. The dress illustrates a consistent rise in average throughput from 94 items per hour at 0.8 worker’s processing rate to 173 items per day at 1.2 worker’s processing rate.

**Fig. 13:**
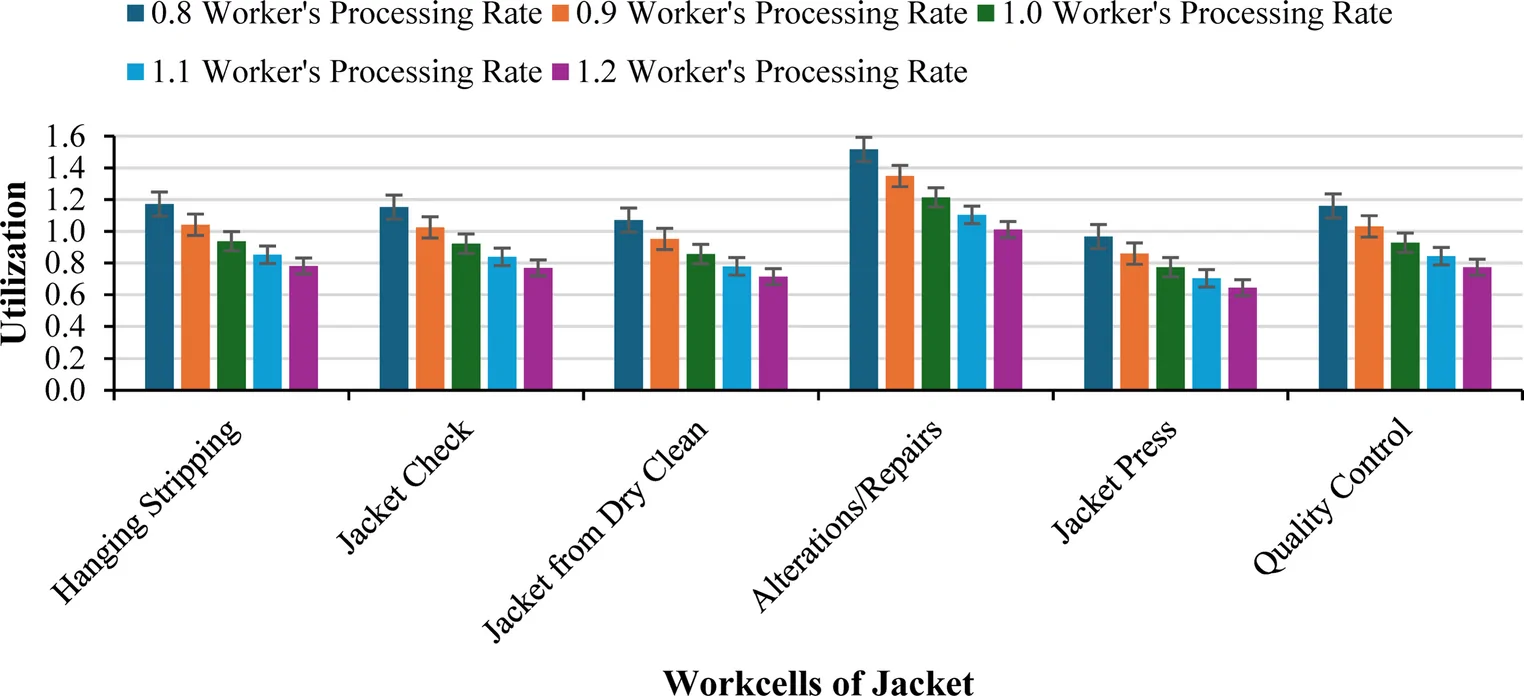
Utilization of the workcells in jacket processing in varying from 0.8 worker’s processing rate to 1.5 worker’s processing rate

The utilization of each workcell, varying from 0.8 to 1.2 worker’s processing rate is shown in Fig. 13. At 0.8 and 0.9 worker’s processing rates, the utilization of each workcell exceeds 1, signifying a garment queue at the associated workcell. Consequently, the average throughput is 809 and 911 items per hour, with median lead times of 6.49 h and 6.25 h, respectively. Further increase in the worker’s processing rate from 1 to 1.2 initially raised the average throughput to 1013 items per day at a worker’s processing rate of 1 but subsequently stabilized at an average throughput of 1010 items per day at worker’s processing rates of 1.1 and 1.2, with a decrease in the median lead time from 6.16 h to 5.93 h.

**Fig. 14:**
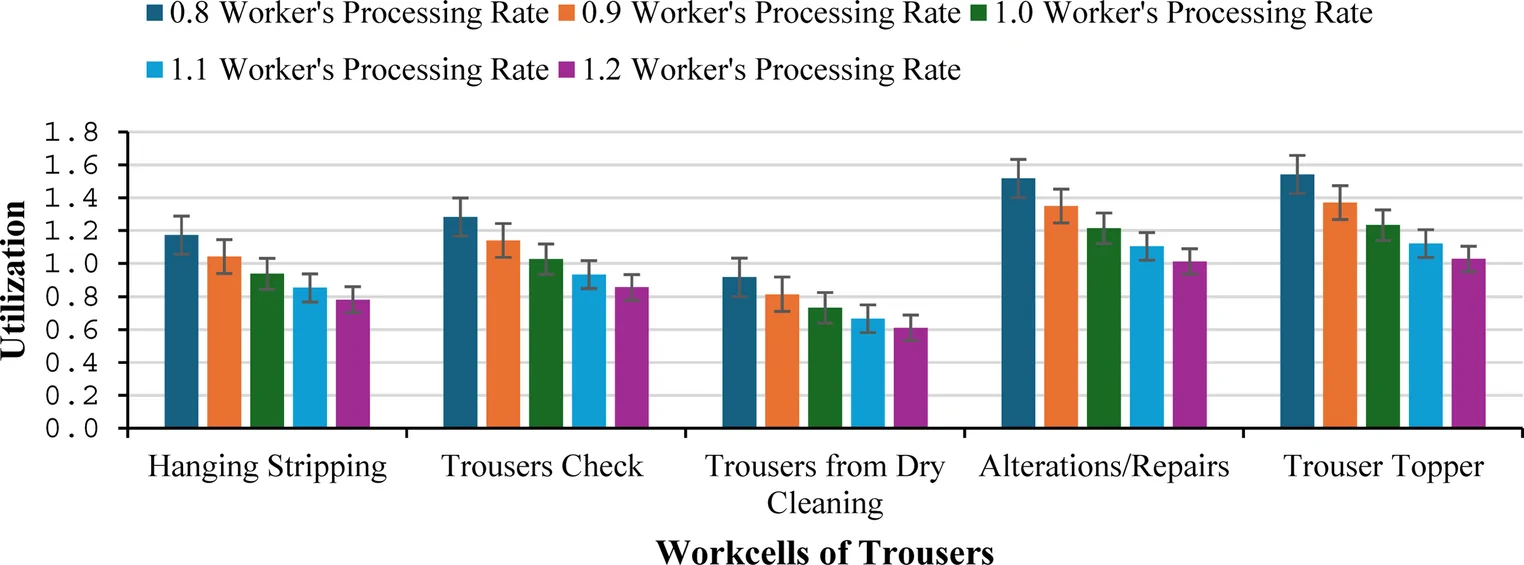
Utilization of the workcells in trousers processing in varying from 0.8 worker’s processing rate to 1.5 worker’s processing rate

The workcells used for trousers are depicted in Fig. 14. For 0.8 and 0.9 worker’s processing rates, all workcells (except Trousers from dry cleaning), exhibit workcell utilization between 1 and 1.5. Indicating a backlog of trousers, the average throughput is 532 and 599 items per day, with median lead times of 6.35 and 6.24 h, respectively. With an improvement in the worker’s processing rate from 1 to 1.2, the average throughput steadily increased from 564 items per day at a 1 worker’s processing rate to 749 items at a 1.2 worker’s processing rate, resulting in a decrease in lead time from 6.14 h to 5.86 h.

**Fig. 15:**
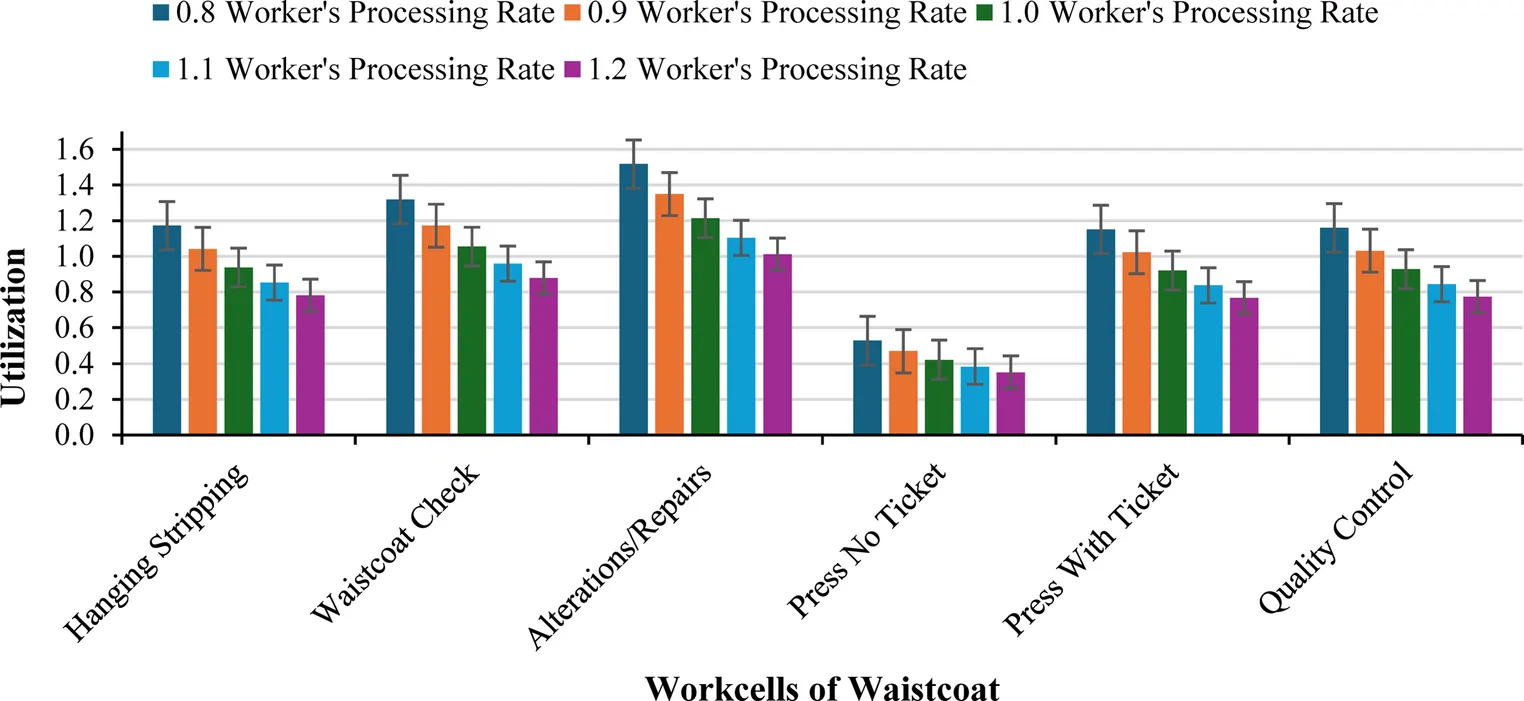
Utilization of the workcells in waistcoat processing in varying from 0.8 worker’s processing rate to 1.5 worker’s processing rate

Figure 15 illustrates the workcell use for the waistcoat. The utilization rate of each workcell at worker’s processing rates of 0.8, 0.9, and 1 is below 1, reflecting a steady improvement in average throughput to 384, 468, and 538 items per day, supported by a reduction in median average time from 5.77 h to 5.64 h. However, further increase of worker processing rates from 1 to 1.1–1.2 reveals a significant rise in average throughput at 1.1 (527 items per day), while at 1.2 worker’s processing rates, the average throughput stays constant. The workcell utilization decreased to 0.7 to 0.8 at the high worker’s processing rate. The median lead time exhibits a declining trend as worker processing rates increase.

**Fig. 16:**
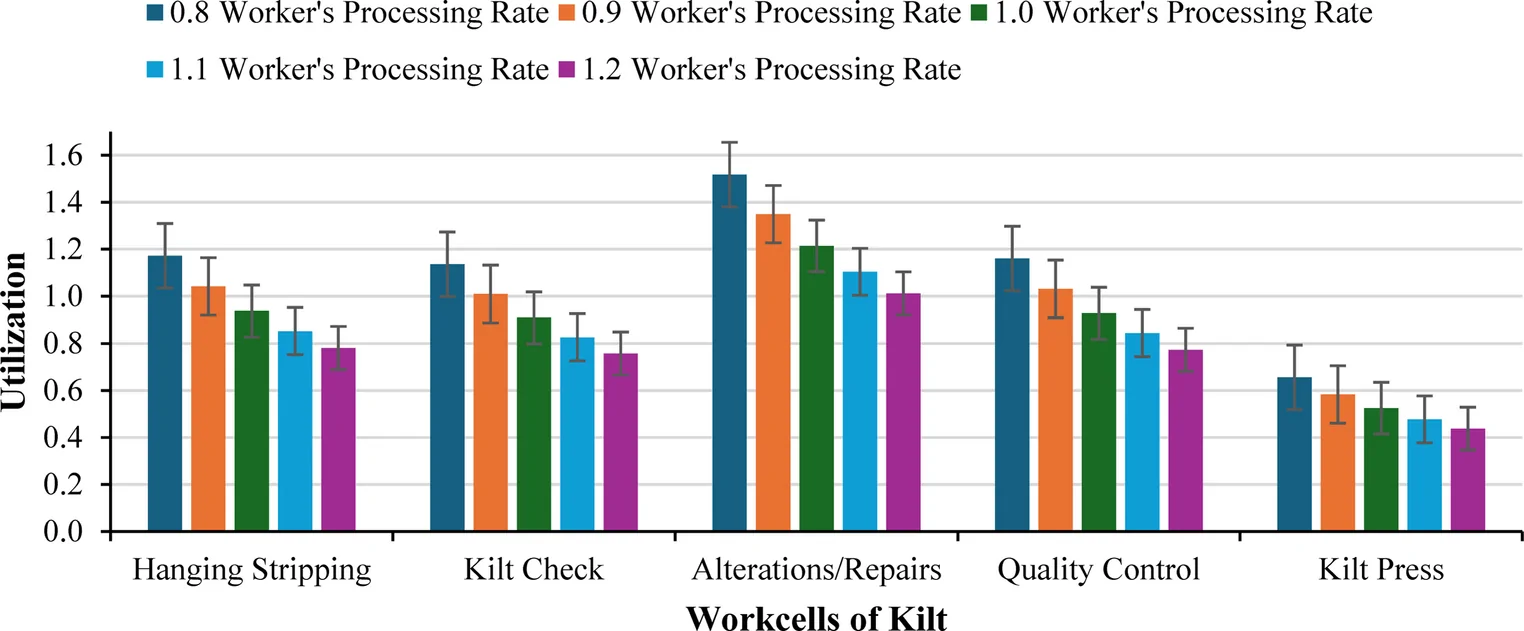
Utilization of the workcells in kilt processing in varying from 0.8 worker’s processing rate to 1.5 worker’s processing rate

Figure 16 illustrates the decomposed workcell usage for each kilt workcell. At a worker processing rate of 0.8, the utilization of each kilt workcell exceeds 1 (except the kilt press). The situation has led to a backlog at the workcell, resulting in an average throughput of 114 items per day and a median lead time of 8.66 h. With a further rise in the worker’s processing rate to 0.9, 1, and 1.1, the utilization of each workcell remains below 1, demonstrating a positive trend in average throughput, yielding 137, 145, and 151 items per day, respectively. However, a further increase to a 1.2 worker processing rate has no meaningful effect on the average throughput, resulting in no gain in average throughput.

**Fig. 17:**
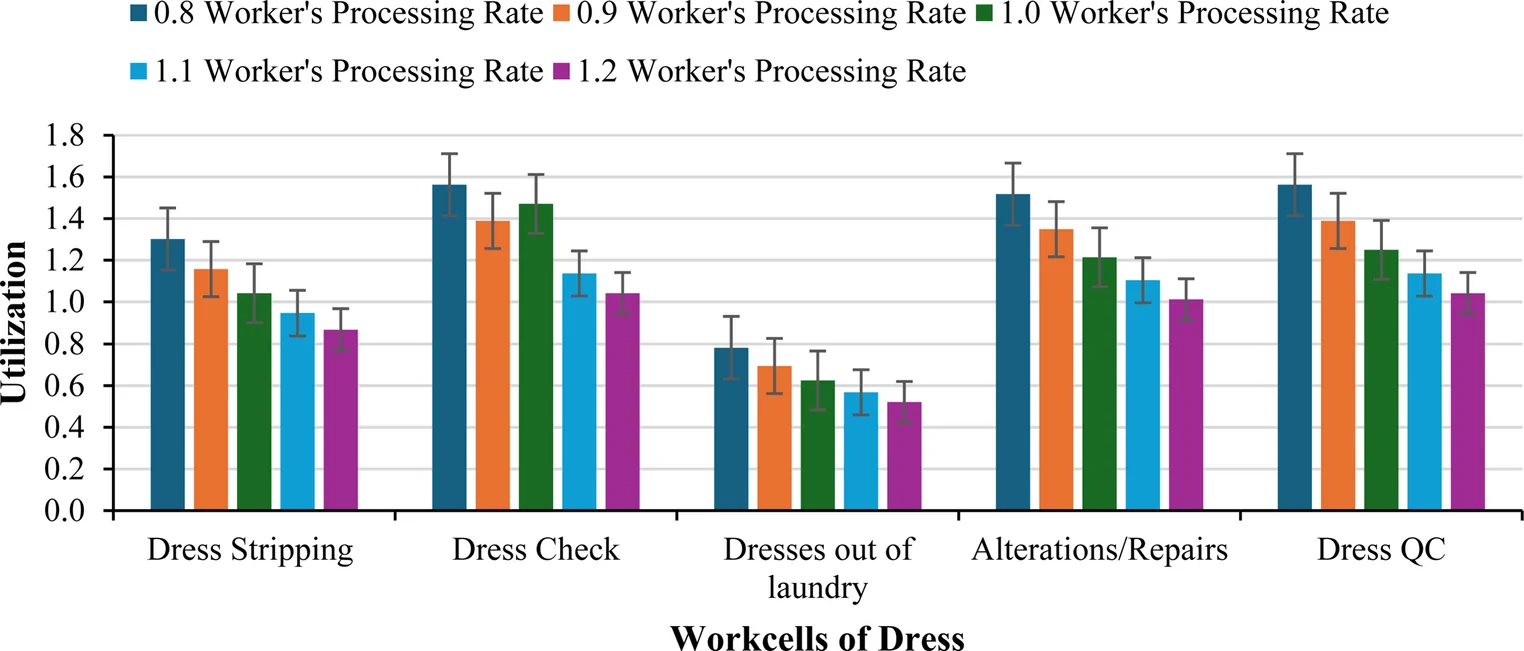
Utilization of the workcells in dress processing in varying from 0.8 worker’s processing rate to 1.5 worker’s processing rate

Figure 17 illustrates the workcell usage for dresses at different worker processing rates. The workcell utilization decreases from 1.5 to 0.9 at a processing rate of 0.8 to 1.2 workers, (excluding dresses from washing having utilization less than 1). The data indicates a consistent enhancement in average throughput, commencing at 94 items per day with a worker’s processing rate of 0.8, and progressively escalating to 115 items (at 0.9 worker’s processing rate), 136 items (at 1 worker’s processing rate), 158 items (at 1.1 worker’s processing rate), and 173 items (at 1.2 worker’s processing rate) per day. The median lead time decreases from 3.95 h with 0.8 worker’s processing rate to 3.53 h at a 1.2 worker’s processing rate.

### Discussion

The tradeoff curves for multiple garment types in Fig. 5 illustrate how the arrival rate impacted each garment throughput. As the arrival rate rises from 0.8 to 0.9, the throughput of jackets, waistcoats, and kilts increases, but the throughput of trousers and dresses declines. At an arrival rate of 1, the waistcoat and kilt exhibited their greatest average throughput across all ranges of arrival rates. The jacket exhibited enhanced throughput, but the throughput of pants and dresses decreased. At an arrival rate of 1.25, the jacket exhibited its peak average throughput across all arrival rate ranges. All other garments had a reduced average throughput in comparison to the first arrival rate. An additional rise in the arrival rate to 1.5 resulted in a decline in the average throughput of all garments. Consequently, the tradeoff analysis offers critical insights on the selection of arrival rates to achieve the necessary throughput for a certain garment type.

Figure 12 demonstrates the tradeoff among several garment types, highlighting the impact of the worker’s processing rate on the throughput of each garment type. At a worker’s processing rate of 0.8, all garment types exhibited their lowest average throughput across the whole spectrum of processing rates. At 0.9 processing rate, all garments exhibit a positive increase in average throughput. The jacket and waistcoat exhibited a significant increase. At 1 worker’s processing rate, the jacket and kilt exhibited their peak average throughput over the whole spectrum of processing rates. The throughput of other clothing types also increased. At 1.1 worker’s processing rate, the average throughput of jackets and kilts remains constant. However, the throughput for trousers, waistcoats, and dresses has risen. At 1.2 worker’s processing rate, the average throughput for waistcoats remained constant. However, the average throughput for trousers and dresses rose. Consequently, the tradeoff curves provide insights into the average throughput influenced by the worker’s processing rate.

The external arrival rate and the worker’s processing rate significantly affect the lead time and throughput of each garment type. A decrease in the arrival rate results in network underutilization, whereas an increase over a given threshold leads to queue accumulation at the workcell due to overutilization. A low worker’s processing rate will reduce throughput, whereas an increase in worker’s processing rate will enhance network performance up to a certain limit, beyond which it will stabilize. Consequently, with fluctuation in arrival rate and worker’s processing rate in the network, the tradeoff analysis will be helpful to optimize the throughput of the garment type. Given the constraint of limited resources, rearranging workers within the network for optimal workcell utilization positively influences throughput and lead time. The following section will examine how rearranging workers with identical resource capacity can improve network utilization.

## Rearrangement of workers based on the utilization of the workcell

**Fig. 18:**
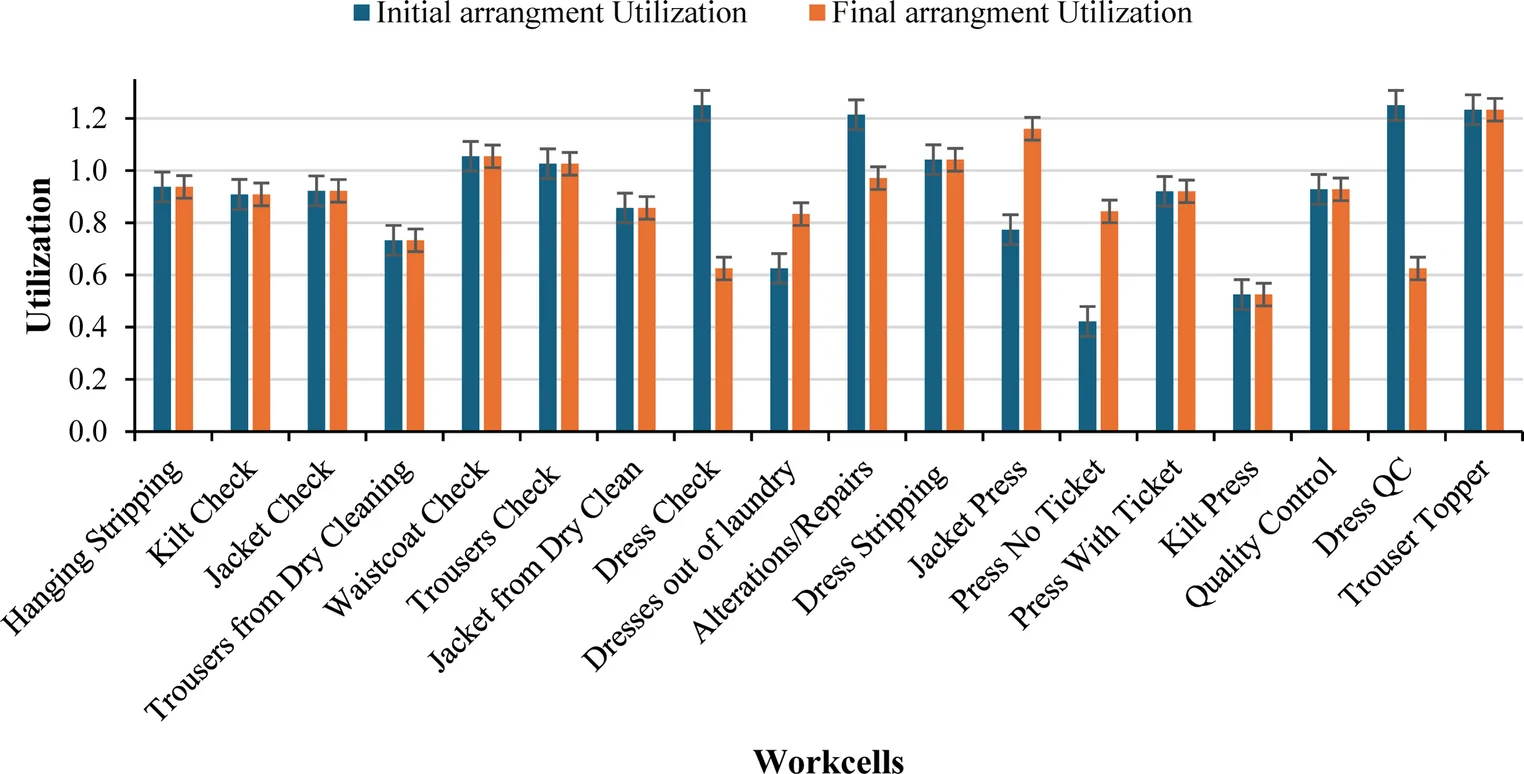
Initial and final arrangement utilization of each workcell

Table 7 illustrates the initial arrangement of workers and each worker’s processing rate at each workcell. The external arrival rate at the first point of the network model is 675 items per hour for hanging stripping and 25 items per hour for dress stripping. The initial utilization of each workcell is shown in Fig. 18 considering this arrangement.

**Table 7:** Initial arrangement and final rearrangement of workers at each workcell

Workcell	Worker’s Processing rate (Items/hour)	Initial arrangement	Final arrangement
Hanging Stripping	90	8	8
Kilt Check	13	2	2
Jacket Check	23	7	7
Trousers from Dry Cleaning	14	5	5
Waistcoat Check	48	2	2
Trousers Check	25	5	5
Jacket from Dry Clean	26	2	2
Dress Check	20	1	2
Dresses out of laundry	10	4	3
Alterations/Repairs	8	4	5
Dress Stripping	12	2	2
Jacket Press	16	3	2
Press No Ticket	12	2	1
Press With Ticket	22	2	2
Kilt Press	9	1	1
Quality Control	60	4	4
Dress QC	20	1	2
Trouser Topper	26	4	4

The initial utilization rates for dress check, alteration/repair, and dress quality control are 1.25, 1.22, and 1.25, respectively. Conversely, the utilization rates for jacket press, press without tickets, and dresses out of wash are 0.77, 0.42, and 0.63, respectively. For all other workcells, the utility ranges from 0.9 to 1.05. The over usage of the workcell often leads to increased queues, hence diminishing throughput, while underutilized workcells with the ideal time result in a decline in throughput. Therefore, overutilized and underused workcells need the reallocation of workers to achieve line balance.

At the dress check, alteration/repair, and dress QC. there is an addition of one worker. Conversely, at the jacket press, press no ticket, and dresses out of laundry, one worker is reduced. This rearrangement results in the final utilization of previously over-utilized workcells as follows: dress check 0.63, alteration/repair 0.97, and dress QC 0.63. For the under-utilized workcells, the utilization rates are dresses out of laundry 0.83, jacket press 1.16, and press no ticket 0.84.

The average throughput consequent to rearrangement is shown in Table 8. The arrangement had a significant favourable impact on the average throughput of dresses. There is considerable enhancement in the throughput of waistcoats and kilts, whereas there is a slight improvement for jackets and trousers. The rearranging of workcells effectively utilizes workcell alteration/repair, is beneficial to all garments, and positively influences the average throughput of all garment types. The rearranging of dress check, dress QC, and dresses out of laundry has positively influenced the average throughput of dresses. The usage of the jacket press has marginally increased to 1, which may have a mild impact on the average throughput of jackets. The rearrangement at Press no ticket, the utilization remains below 1, therefore having no adverse impact on the average throughput of the waistcoat. However, the enhancement in worker’s processing rates at the trousers topping and jacket check workcells might improve the throughput of both trousers and jackets.

**Table 8:** Average throughput in the initial arrangement and final rearrangement of each garment type

Garment types	Average throughput per day	
	Initial arrangement	Final arrangement
Jacket	1013	1019
Trousers	602	614
Waistcoat	538	551
Kilt	154	167
Dress	137	164

## Model validation

**Fig. 19:**
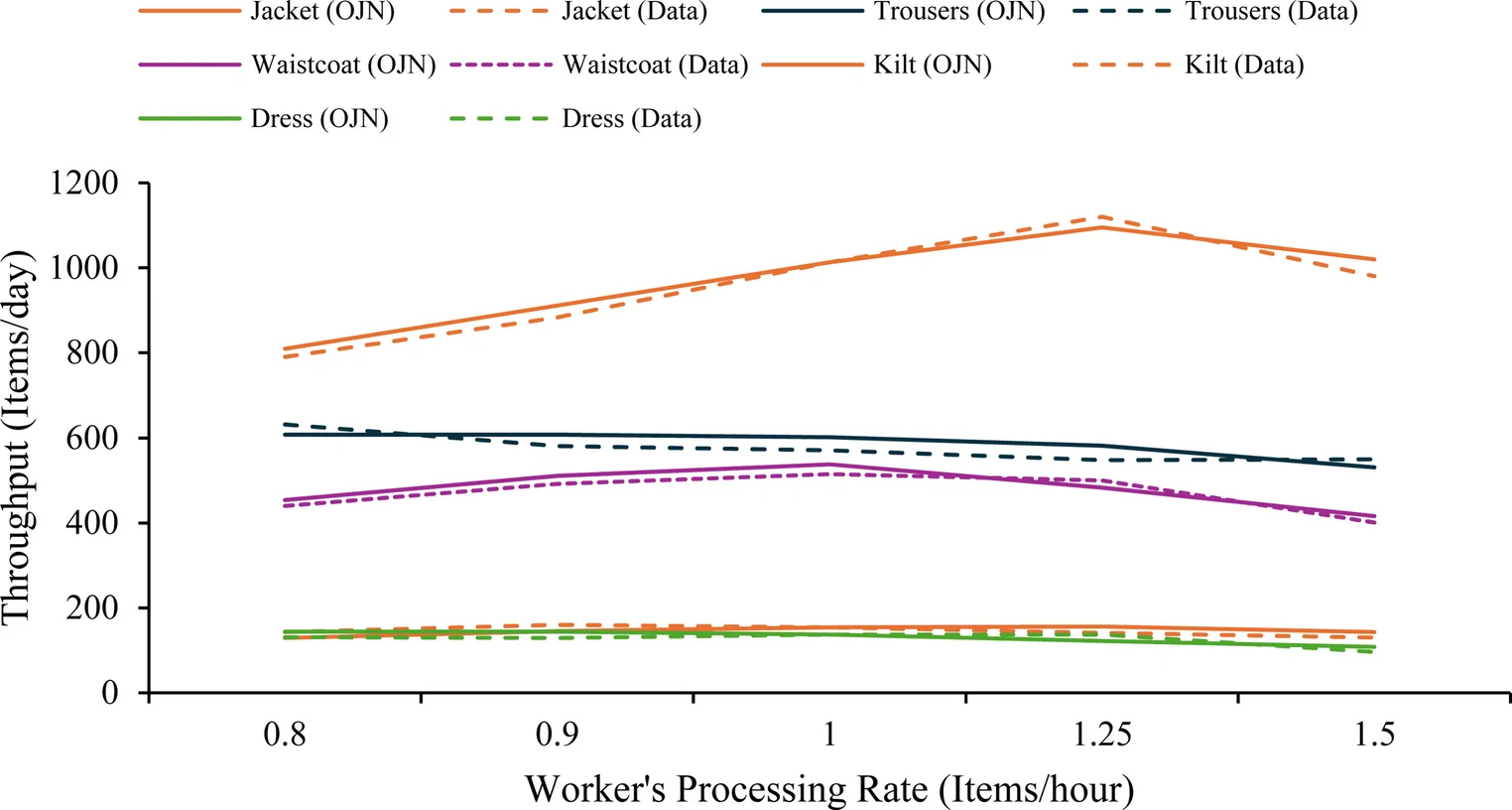
Comparison of throughput between the open Jackson queuing network (OJN) model and actual industry values (Data) for the worker’s processing rate

**Fig. 20:**
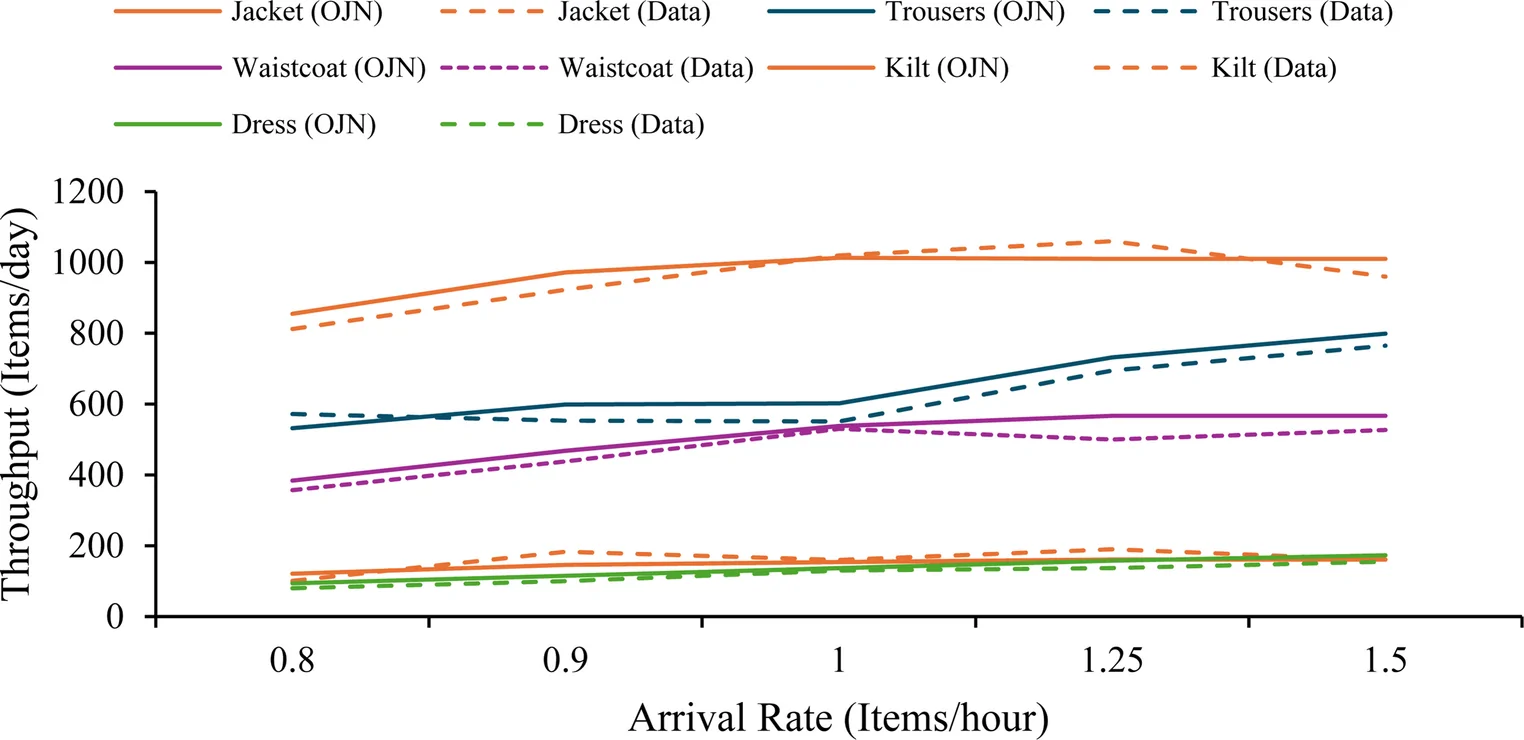
Comparison of throughput between the open Jackson queuing network (OJN) model and actual industry values (Data) for the arrival rate

The open Jackson queuing network is a complex method that can be simplified by assumptions. However, these assumptions may not precisely reflect the real system characteristics. In these circumstances, the real-world data are utilised to validate the precision of the proposed approach. Fig. 19 shows the comparison of the throughput of different garment types for worker’s processing rate, while Fig. 20 shows the comparison of the throughput of different garment types for arrival rate. The average daily data of the year obtained from the garment reprocessing industry is used to compare the results.

The results comparison revealed a significant alignment between the open Jackson queueing network estimations and the real-world data set. The tradeoff results are very close to the actual values, with minor deviations. The deviation is within ± 3 to 7% of the real-world dataset. These deviations are attributed to the assumption in the open Jackson queuing network. However, adjustments can improve accuracy, making it a more robust production planning and decision-making tool.

To ensure the validation of the open Jackson Queueing network model, structured interviews were conducted with production managers (03), line supervisors (05), and operations staff (20) of the garment reprocessing facility. The interviews with industry professionals aim to obtain insights into actual processing times, throughput rates, and factors influencing the efficiency of garment processing. This information will support the validation of the accuracy and reliability of the established open Jackson network model for evaluating lead time and throughput in garment reprocessing. The objective of the interview was to collect feedback on real-world information on perceived lead times, throughput expectations, resource constraints, and flow consistency among garment types, which could be matched to the analytical predictions of the model, with a particular emphasis on evaluating whether the model precisely reflects actual conditions of garment reprocessing operations. This interview collected feedback on apparent lead times, expected average throughput, resource/worker constraints, and transit flow of different garment types.

The interview has four parts. Part-A: Workcell processing times and efficiency includes a survey about the average processing time of different garment types at each workcell. Factors affecting the processing time variation, worker allocation at different workstations, and a survey of the effect of worker’s level of experience that affect the processing rates of various garment types on different workcells. The interview feedback showed that workers are categorized into beginner/intermediate/expert levels. The worker’s processing rate is high with expert workers, and it can be improved for beginners and intermediate workers by following the standard operation process approach and by continuous training in processing. The customer’s quality of the returned garment affects the additional reprocessing time and results in increased processing time. The expected garments return from the customers helps to allocate workers to workcells.

Part B: Transit time between workcells. This part includes feedback on the transit time of garment types between each workcell, factors affecting transit delay, and buffer area waiting time for different garment types. In the survey, participants confirmed that jackets transferred faster than other garments due to a fully automatic transfer line, followed by a kilt and a waistcoat with a semi-automatic transfer line. At the same time, trousers and dresses have manual transfer only. All workcells have buffer storage, and based on the garment priority, availability of the subsequent workcell, and availability of workers to process the garment, the transit time of the garment varies from a few minutes to a couple of hours. Part-C: Production flow and bottleneck include the survey of challenges in process efficiency and strategies for resource allocation to workcells to reduce lead process time and increase throughput of various garment types. The feedback from participants showed that the worker’s processing rate, garment arrival rate, and garment defect rate highly affect the garment processing efficiency. As most garment reprocessing is labor-intensive, the workers’ processing rate affects the overall throughput. The arrival rate and transit between the workcell control the congestion, which affects the waiting time and lead time. The garment with fewer defects is processed faster compared to a more defective garment, which affects overall processing efficiency. The limited workcell capacity and garment priority have a moderate effect on garment reprocessing and can be controlled by effective worker allocation. Due to fewer machines involved, machine breakdown has the least impact. Due to the garment flow forecasted from the customer’s order, effective resource allocation can be done in advance, hence, poor production planning has the least effect on garment processing time and throughput.

Part-D: Validation and comparison with the open Jackson queueing network model. This part includes the approximate lead production time and the daily throughput of various garment types per day. This part of the interview observes the model’s limitations in capturing real-world factors and the model’s effectiveness for decision-making in process planning of different garment types for daily throughput. The feedback revealed that reported lead time analyses for different garment types are identical to the estimated model, and frequent delay workcells have appeared to be causes of processing delay. Most participants confirmed that the mathematical queuing structure implemented in the framework accurately reflected realistic factory floor dynamics, offering robust qualitative validation of the open Jackson queueing network as a dependable analytical approach.

## Conclusions and future work

This research aimed to investigate the management of throughput variations of various garment types and the key trade-off considerations inside a garment reprocessing network operating in a customer-order-driven market. The customers’ needs fluctuate throughout the year. To meet the customer rental demand with profit in business, a tradeoff decision among the garment types must be addressed. In this paper, we explore the application of tradeoff curves in the rental garment reprocessing network, which was a novel investigation attempt. We focused on a complex network expressed as an open Jackson queueing network and a decomposition approach to analyze the performance characterization of system utilization. We performed a sensitivity analysis emphasizing the lead time and throughput of each garment type by varying the external arrival rate and the worker’s processing rate. The increase in external arrival rate improves the average throughput of jackets, waistcoats, and kilts to a certain point, beyond which it begins to decline, but the average throughput for trousers and dresses consistently decreases. The rise in external arrival rate increased the median lead time for each garment type. The average throughput stabilized after a specific increase in the worker’s processing rates for jackets, waistcoats, and kilts. It demonstrated a favourable enhancement in average throughput for both trousers and dresses. The median time decreases as the worker’s processing rate increases for each garment type. The rearranging of workers in the network workcell improves the average throughput of each garment type. The study provides extensive insights for a customer order-driven rental garment reprocessing business model, including a trade-off analysis of lead time and throughput for various garment types. It emphasizes the need to manage performance metrics and related trade-off attributes, concentrating on the throughput of reprocessing rental garment types. Subsequently, this strategy aids the rental garment business model in organizing specific garment inventory controls.

This investigation has specific limitations that must be addressed in future studies. The costs associated with operating each workcell and the variability of process parameters will provide a deeper understanding of the tradeoff support between various garment types. This implies the potential for a comprehensive decision-making approach to integrate performance and cost analysis. Implement finite buffer storage for each workcell while examining the system weekly or monthly to assess the impact on queue length, waiting time, and throughput changes. The dynamic allocation of workers according to the workcell usage and bottlenecks using a queue network model to analyse variations in lead process time and throughput across different garment types.
